# Myosin and tropomyosin–troponin complementarily regulate thermal activation of muscles

**DOI:** 10.1085/jgp.202313414

**Published:** 2023-10-23

**Authors:** Shuya Ishii, Kotaro Oyama, Fuyu Kobirumaki-Shimozawa, Tomohiro Nakanishi, Naoya Nakahara, Madoka Suzuki, Shin’ichi Ishiwata, Norio Fukuda

**Affiliations:** 1 Foundational Quantum Technology Research Directorate, National Institutes for Quantum Science and Technology, Gunma, Japan; 2 Department of Cell Physiology, The Jikei University School of Medicine, Tokyo, Japan; 3 Department of Anesthesiology, The Jikei University School of Medicine, Tokyo, Japan; 4 Department of Molecular Physiology, The Jikei University School of Medicine, Tokyo, Japan; 5 Institute for Protein Research, Osaka University, Osaka, Japan; 6 Department of Physics, Faculty of Science and Engineering, Waseda University, Tokyo, Japan

## Abstract

Contraction of striated muscles is initiated by an increase in cytosolic Ca^2+^ concentration, which is regulated by tropomyosin and troponin acting on actin filaments at the sarcomere level. Namely, Ca^2+^-binding to troponin C shifts the “on–off” equilibrium of the thin filament state toward the “on” state, promoting actomyosin interaction; likewise, an increase in temperature to within the body temperature range shifts the equilibrium to the on state, even in the absence of Ca^2+^. Here, we investigated the temperature dependence of sarcomere shortening along isolated fast skeletal myofibrils using optical heating microscopy. Rapid heating (25 to 41.5°C) within 2 s induced reversible sarcomere shortening in relaxing solution. Further, we investigated the temperature-dependence of the sliding velocity of reconstituted fast skeletal or cardiac thin filaments on fast skeletal or β-cardiac myosin in an in vitro motility assay within the body temperature range. We found that (a) with fast skeletal thin filaments on fast skeletal myosin, the temperature dependence was comparable to that obtained for sarcomere shortening in fast skeletal myofibrils (*Q*_10_ ∼8), (b) both types of thin filaments started to slide at lower temperatures on fast skeletal myosin than on β-cardiac myosin, and (c) cardiac thin filaments slid at lower temperatures compared with fast skeletal thin filaments on either type of myosin. Therefore, the mammalian striated muscle may be fine-tuned to contract efficiently via complementary regulation of myosin and tropomyosin–troponin within the body temperature range, depending on the physiological demands of various circumstances.

## Teaser

Ishii et al. analyze the microheating-induced sliding movements of reconstituted thin filaments in an in vitro motility assay. They find that the temperature dependence of thin filament sliding is complementarily regulated by myosin and tropomyosin–troponin within the body temperature range.

## Introduction

Upon sarcolemmal depolarization, contraction of striated muscle, either skeletal or cardiac, is triggered by an increase in the intracellular Ca^2+^ concentration ([Ca^2+^]_i_). This Ca^2+^-dependent mechanism is regulated by the regulatory proteins tropomyosin (Tm) and troponin (Tn) on filamentous actin (F-actin; e.g., Ebashi and Endo, 1968; Gordon et al., 2000; Kobirumaki-Shimozawa et al., 2014). In both skeletal and cardiac muscle, when [Ca^2+^]_i_ is low under the relaxing condition (at pCa (= −log [Ca^2+^]) ≥7), the Tm–Tn complex blocks the binding of myosin to actin (“off” state). However, upon an increase in [Ca^2+^]_i_, the binding of Ca^2+^ to the regulatory Ca^2+^-binding site(s) of TnC leads to displacement of Tm on F-actin (“on” state). This conformational change of thin filaments allows myosin to interact with actin, and accordingly, active force is generated. It is likewise well established that the formation of strongly bound crossbridges (i.e., rigor crossbridges or ADP-bound crossbridges) promotes the binding of neighboring myosin to thin filaments, shifting the on–off equilibrium of the thin filament state toward the on state in both skeletal and cardiac muscle (see Shimizu et al., 1992; Fukuda et al., 1998, 2000; Kobirumaki-Shimozawa et al., 2014 and references therein). Therefore, Ca^2+^ and strongly bound crossbridges activate thin filaments in a complementary manner in both types of muscle.

Ambient temperature is an important factor that affects myofibrillar Ca^2+^ sensitivity in both skeletal and cardiac muscles (e.g., Mühlrad and Hegyi, 1965; Murphy and Hasselbach, 1968; Hartshorne et al., 1972; Stephenson and Williams, 1981; Harrison and Bers, 1989). It has been reported that heating elicits myofibrilar force generation in a Ca^2+^-independent manner (for review see Ranatunga, 2018; Ishii et al., 2020). For example, heating has been shown to increase active force in intact frog sartorius muscle (Hill, 1970) and skinned rabbit psoas fibers (Ranatunga, 1994) in a relaxing solution. More recently, we demonstrated that a rapid increase in temperature (within 0.2 s) via water-absorbable 1,455-nm infrared (IR) laser irradiation causes reversible and reproducible shortening of intact and skinned (Triton X-100-treated) isolated rat cardiomyocytes (Oyama et al., 2012). Measurement of [Ca^2+^]_i_ revealed that this thermal activation is not preceded by intracellular Ca^2+^ transients, unlike normal excitation–contraction coupling, hence it is regulated in a Ca^2+^-independent manner. A similar finding was reported by Marino et al. (2017), who demonstrated that optical heating of gold nanoparticles induces Ca^2+^-independent contraction in mouse C2C12 myotubes. Moreover, studies taking advantage of an in vitro motility assay showed that increasing temperature induces sliding movements of reconstituted thin filaments in the absence of Ca^2+^ (pCa 9), above 35°C with human cardiac Tm and bovine cardiac Tn (Ishii et al., 2019a) and above 43°C with human cardiac Tm and human cardiac Tn (Brunet et al., 2012). These findings obtained under various experimental conditions are in agreement with the supposition that crossbridge cycling is accelerated by heating in a Ca^2+^-independent manner (see Ishii et al., 2019a, 2020 for details).

An early solution study showed that the Tm–Tn complex dissociates from F-actin at greater than ∼45°C in the absence of Ca^2+^ (Ishiwata, 1978; proteins extracted from rabbit fast skeletal muscle). It is therefore likely that Ca^2+^-independent thermal activation occurs at least in part via partial dissociation of Tm–Tn from F-actin, promoting actomyosin interaction (as discussed in Ishii et al., 2019a, 2020). However, it is possible that the temperature-dependent activation of striated muscle is regulated not only by Tm–Tn but also by myosin. Indeed, Caremani et al. (2019) reported that lowering the temperature from near physiological 35°C down to 10°C in mouse skeletal muscle traps myosin heads in a refractory state, which prevents their binding to thin filaments and causes a marked decrease in tetanic force.

To elucidate the molecular mechanisms of the temperature-dependent activation of striated muscle, in the present study, we investigated the effects of heating via IR laser irradiation on sarcomere dynamics along isolated fast skeletal myofibrils. Then, we analyzed the temperature dependence of the heating-induced sliding of fast skeletal or cardiac Tm–Tn-reconstituted thin filaments on fast skeletal or β-cardiac myosin in an in vitro motility assay. We examined four combinations of experimental data at pCa 9 and 5, i.e., (1) skeletal thin filaments and skeletal myosin, (2) skeletal thin filaments and β-cardiac myosin, (3) cardiac thin filaments and skeletal myosin, and (4) cardiac thin filaments and β-cardiac myosin. In doing so, we yielded a conclusion regarding the differential action of heating on the skeletal versus cardiac contractile systems. Namely, our findings show that the lower thermal activation of fast skeletal thin filaments is compensated by the higher thermal activity of fast skeletal myosin, and the higher thermal activation of cardiac thin filaments is compensated by the lower thermal activity of β-cardiac myosin. We discuss the mechanistic implications based on the complemental thermal regulation of the contractile system by myosin and Tm–Tn.

## Materials and methods

Purification of proteins was performed at Waseda University, in accordance with the Guidelines for Proper Conduct of Animal Experiments approved by the Science Council of Japan and the “Regulations for Animal Experimentation” of Waseda University. Preparation of fast skeletal myofibrils and microscopic observations were performed at The Jikei University School of Medicine, in accordance with the “Guidelines on Animal Experimentation of The Jikei University School of Medicine.” The study protocol was approved by the “Animal Care Committee of The Jikei University School of Medicine.”

### Preparation of fast skeletal myofibrils

Male adult white rabbits (∼2−3 kg) were purchased from Sankyo Labo Service and anesthetized with 3−4% isoflurane (Pfizer) inhalation followed by an intravenous administration of 50 mg/kg pentobarbital sodium (NACALAI TESQUE). Then, the animals were immediately euthanized by exsanguination. Muscle strips (∼1−2 mm in diameter and ∼10 mm in length) were dissected from psoas muscles in Ca^2+^-free Tyrode’s solution containing 30 mM 2,3-butanedione monoxime (BDM; see Terui et al., 2008; Matsuba et al., 2009 for composition). The muscles were skinned in relaxing solution (5 mM ATP [Sigma-Aldrich], 1 mM Mg^2+^, 10 mM ethylene glycol bis [β-aminoethyl ether] N,N′-tetraacetic acid [EGTA; Sigma-Aldrich], 1 mM dithiothreitol [DTT; Nakalai Tesque], 40 mM N,N-Bis(2-hydroxyethyl)-2-aminoethanesulfonic acid [Sigma-Aldrich], 15 mM phosphocreatine [Sigma-Aldrich], 15 U/ml creatine phosphokinase [Sigma-Aldrich]) containing 1% (wt/vol) Triton X-100, and 10 mM BDM overnight at ∼3°C (Fukuda et al., 2003). Ionic strength was adjusted to 180 mM by K-propionate (Tokyo Chemical Industry) and pH to 7.0. Muscles were stored for up to 3 wk at −20°C in a relaxing solution containing 50% (vol/vol) glycerol.

Myofibrils were prepared by homogenizing the fibers in a relaxing solution based on our previously published procedure (Shimamoto et al., 2007). Myofibrils were stored on ice and used within 5 h. The relaxing solution, with or without glycerol, contained protease inhibitors (0.5 mM phenylmethylsulfonyl fluoride, 0.04 mM leupeptin, 0.01 mM E64) to avoid protein degradation.

### Purification of proteins

Human α-Tm for the reconstitution of cardiac thin filaments was expressed in *E. coli* and purified in the laboratory of Dr. Bryant Chase (Florida State University, Tallahassee, FL; as in Schoffstall et al., 2006; Wang et al., 2011). Cardiac Tn (complex of TnT, TnI, and TnC) was extracted from fresh bovine ventricles in the laboratory of Dr. Masataka Kawai (University of Iowa, Iowa City, IA; as in Potter, 1982). β-Cardiac myosin was extracted from fresh porcine ventricles in the laboratory of Dr. Motoshi Kaya (University of Tokyo, Tokyo, Japan; as in Harada et al., 1990; Schoffstall et al., 2006).

The Tm–Tn complex, actin, and skeletal heavy meromyosin (HMM: α-chymotrypsin proteolytic fragment of myosin II) were purified from rabbit fast skeletal muscles (as in Fujita et al., 1996; Suzuki et al., 1996). For purification of these proteins, male adult white rabbits (∼2−3 kg) were purchased from Japan Laboratory Animals and sacrificed based on a previously published procedure (Suzuki and Ishiwata, 2011). We used fast skeletal actin throughout the present study because actin is one of the most conserved proteins known, and rabbit fast skeletal actin, which is easy to obtain in a large quantity, differs from human cardiac actin by only four conservative residue changes (see NCBI GeneBank IDs XP_002722940.2 and NP_005150.1 for rabbit skeletal actin and human cardiac actin, respectively).

### Solutions for heating experiments

The experiments with fast skeletal myofibrils were performed in a relaxing solution (see above for composition). The compositions of solutions used for an in vitro motility assay were as follows: (a) F-buffer: 2 mM MgCl_2_, 1.5 mM NaN_3_, 100 mM KCl, 10 mM DTT, 2 mM 3-(N-morpholino) propane sulfonic acid and pH 7.0; (b) Rigor solution (pCa 9): 4 mM MgCl_2_, 1 mM EGTA, 25 mM KCl, 10 mM DTT, and 25 mM imidazole-HCl (Im-HCl); (c) Relaxing (non-activating) solution (pCa 9): 4 mM MgCl_2_, 1 mM EGTA, 25 mM KCl, 10 mM DTT, and 25 mM Im-HCl; and (d) Activating solution (pCa 5): 1 mM EGTA, 1 mM CaCl_2_, 4 mM MgCl_2_, 25 mM KCl, 10 mM DTT, and 25 mM Im-HCl. For rigor, relaxing, and activating solutions, the ionic strength was adjusted to 50 mM by KCl, and pH to 7.4 by KOH. Relaxing and activating solutions containing 2 mM MgATP were used for heating experiments, hence termed experimental solutions (see below for details). The pCa of solutions was calculated by using the computer program in Wang and Kawai (2001). Temperature-dependent changes in the affinity of EGTA for Ca^2+^ (as in Harrison and Bers, 1987) had minimal effects on the free Ca^2+^ concentration in either relaxing (pCa 9) or maximally activating (pCa 5) solution. Chemicals were purchased from FUJIFILM Wako Pure Chemical Industries unless otherwise noted.

### Reconstitution of thin filaments

Cardiac and fast skeletal thin filaments were reconstituted in accordance with our previously published procedure (Ishii et al., 2018). In brief, actin was polymerized in F-buffer and labeled with rhodamine-phalloidin (Thermo Fisher Scientific). The labeled actin filaments were stored on ice and used within 2 wk. Thin filaments were reconstituted in Eppendorf tubes (F-buffer, 20 μl) that contained labeled actin filaments (corresponding to 1.2 μM actin) in the presence of 1.2 μM Tm and 1.2 μM Tn (for cardiac thin filaments), or 1.2 μM Tm–Tn (for skeletal thin filaments) on ice for ∼1 h. Then, the annealing treatment (45°C, 10 min) was performed to achieve correct head-to-tail interaction between neighboring Tm molecules along F-actin (as in Ishiwata, 1973) and to improve the reproducibility of results. Afterwards, the tubes were stored on ice again for up to 1 wk.

### Optical setup

We used a phase-contrast and fluorescence microscope described in detail in our previous studies (Ishii et al., 2019a; Oyama et al., 2020, 2022). In brief, the optical setup was built around an inverted microscope (IX73; Olympus). The solution was directly heated by focusing the IR laser beam (λ = 1,455 nm, KPS-STD-BT-RFL-1455-05-CO; Keopsys) under the microscope through an objective lens (UPlanFL N Oil Iris Ph3, 60×, N.A. set at 1.25 throughout the present study; Olympus). The on–off of heating was regulated by an IR-LEGO unit (Sigma Koki). The laser power (0.5 W) was adjusted to 40% (high), 25% (medium), or 15% (low) for fast skeletal myofibrils, and 60% (high), 50% (medium), or 15% (low) for an in vitro motility assay by using neutral density filters (AND-30C; Sigma Koki). A Nipkow confocal scanner unit (CSU-X1; Yokogawa Electric Corporation), a dichroic mirror (Di01-T405/488/561; Semrock Optical Filters of IDEX Corp), an emission filter (FF01-617/73), and an electron-multiplying charge-coupled device (EMCCD) camera (iXon Ultra; Andor Technology) were used for observation of phase-contrast images of myofibrils and fluorescence images of Alexa Fluor 555-dextran excited by a 561 nm laser light (HÜBNER Photonics). In an in vitro motility assay, a mercury lamp (BH2-RFL-T3; Olympus), a mirror unit (U-FGWA; Olympus), and EMCCD camera (iXon 3; Andor Technology) were used for observation of the fluorescence of rhodamine. All images were recorded at 30 frames per second (fps) with ANDOR IQ software (Andor Technology).

### Experiments with fast skeletal myofibrils

An 80-μl drop of relaxing solution containing homogenized myofibrils was applied onto a glass bottom dish (AGC Techno Glass); then, a coverslip (18 × 18 mm; Matsunami Glass) was gently placed on the dish to prevent evaporation of the solution. The glass bottom dish with a coverslip was set on our microscope stage. Experiments were performed at 25 ± 1°C.

### In vitro motility assay experiments

An in vitro motility assay was performed in accordance with our previously published procedure (Ishii et al., 2019a) with slight modifications. In brief, flow cells were prepared by using collodion-coated coverslips (for details see Ishii et al., 2019a). The first drop (20 μl) of fast skeletal HMM (30 μg/ml in relaxing solution) or β-cardiac myosin (200 μg/ml in relaxing solution with 0.6 M KCl added to prevent polymerization of myosin) was applied from one side of the flow cell and incubated for 60 s. These concentrations were the same as those used in our previous study (Ishii et al., 2019a). Subsequently, another drop of the fast skeletal HMM or β-cardiac myosin solution (20 μl) was applied from the other side of the flow cell and incubated for 60 s. This incubation time allowed fast skeletal HMM or β-cardiac myosin to attach to the collodion-coated surface of the coverslip. When β-cardiac myosin was used, the high ionic strength solution (containing a high concentration of KCl) was replaced with a relaxing solution. Then, 20 μl of bovine serum albumin (BSA; Sigma-Aldrich) solution (dissolved at 5 mg/ml in the experimental solution, either relaxing or activating) was applied and incubated for 5 min. Finally, 50 μl of the experimental solution containing F-actin or reconstituted thin filaments (diluted by 1:100) and 1 mg/ml BSA were applied to the flow cell. In experimental solutions for the reconstituted thin filaments, 100 nM excess Tm and 100 nM excess Tn were added to ensure that both molecules were bound to F-actin (see Gordon et al., 1997; Suzuki and Ishiwata, 2011). The experimental solutions also contained 25 mM glucose, 0.22 mg/ml glucose oxidase, 0.036 mg/ml catalase as the oxygen scavenger system, and 2 mM Na_2_ATP (Roche Diagnostics). Methylcellulose 4,000 cP (0.4%; Sigma-Aldrich) was further added to the solutions containing β-cardiac myosin to avoid detachment of myosin from thin filaments (Uyeda et al., 1990; Miyata et al., 1995; Gordon et al., 1997). Finally, two open ends of the flow cell were sealed with nonfluorescent enamel and it was placed on the microscope stage. More than two flow cells were used per condition. All flow cells were studied within <45 min to avoid a reduction in the ATP concentration. Experiments were performed at 23 ± 1°C.

### Temperature measurement

Changes in temperature during microscopic heating (denoted by Δ*T*) were measured from changes in fluorescence intensity (F.I.) of fluorescent thermometers. Namely, in experiments with myofibrils, Alexa Fluor 555-dextran (Thermo Fisher Scientific) was used, as described in our previous study (Oyama et al., 2015). In an in vitro motility assay, rhodamine-phalloidin-labeled F-actin was used in accordance with our previous study (Ishii et al., 2019a). The filaments were incubated on fast skeletal HMM in the rigor solution for 30 min at room temperature (23°C), thereby allowing for the formation of tight-binding actomyosin complexes to suppress the detachment of the filaments during heating (Ishii et al., 2019a). By using the predetermined temperature gradient as described above, Δ*T* of myofibrils or reconstituted filaments (or F-actin) during heating was calculated from the distance between the center of the heat source (i.e., focused laser point) and the targeted region.

### Data analysis

**Figure S1.**
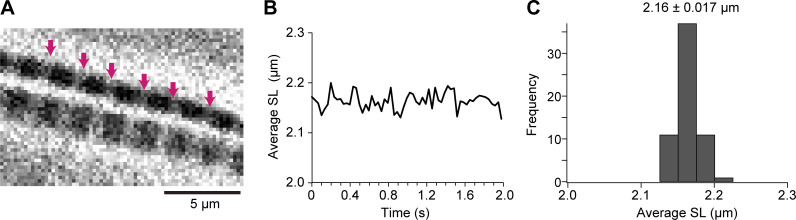
**Fluctuation analysis for the average length of five sarcomeres along a fast skeletal myofibril at rest. (A)** Snapshot of phase-contrast image sequence of two rabbit psoas myofibrils immersed in relaxing solution (pCa 9). Pink arrows indicate six consecutive Z-disks analyzed in B; i.e., the average length of five sarcomeres was analyzed. Imaging was performed at 30 fps. Scale bar, 5 µm. **(B)** Time course of changes in the average SL. **(C)** Histogram showing the variance of SL. Average SL, 2.16 ± 0.017 µm (mean ± SD), indicating that the precision is 17 nm under the present experimental setting.

Myofibrillar shortening experiment: sarcomere length (SL) of myofibrils was analyzed by using the ImageJ software (Schneider et al., 2012) in accordance with our previously published procedure (e.g., Kobirumaki-Shimozawa et al., 2016, 2021). In brief, all data were analyzed by using the multipeak Gaussian fitting program (for details see Kobirumaki-Shimozawa et al., 2016) with Igor Pro 8 (Wavemetrics), and the lengths of individual sarcomeres were calculated as the distance between the centers of two adjacent intensity peaks. Changes in SL (ΔSL) were calculated as SL before heating minus SL during heating. The magnitude of shortening was calculated as ΔSL divided by SL before heating. Fig. S1 shows the fluctuation analysis for the average length of five sequentially connected sarcomeres along a fast skeletal myofibril at rest (pCa 9). The average length and the standard deviation (SD) obtained during the observation time of 2 s was 2.16 ± 0.017 μm at 30 fps, indicating that (a) sarcomeres are at or close to the optimal length (2.24–2.40 μm: full overlap of the thick and thin filaments in rabbit fast skeletal muscle; cf. Yuri et al., 1998) and (b) the precision of the SL analysis under the present experimental setting, at least under the relaxing condition, is 17 nm.

### In vitro motility assay experiment

As detailed in our recent study (Ishii et al., 2019a), we manually tracked the filaments, either F-actin or reconstituted thin filaments, with various time intervals and calculated the sliding velocity in each interval. Then, the data from all intervals were averaged to yield the sliding velocity. The velocity during heating was measured >1 s after the initiation of heating because the temperature reached a plateau within ∼0.5 s (Oyama et al., 2020). Because of the possible presence of local unevenness of the myosin density on the coverslip surface, more than two areas were analyzed in each flow cell and the results were averaged. A mobile filament was defined as a filament that moved >0.4 µm during heating. The threshold was set to 3 × SD of the fluctuation of immobile filaments at pCa 9 before heating. The threshold temperature (*T*_50_) at which 50% of filaments started to move was determined according to the Hill equation:

$$Fraction=\frac{1}{\left\{1+{\left[T_{50}/T\right]}^{h}\right\}}\text{,}$$

where *T* and *h* are temperature and the Hill coefficient, respectively.

The activation energy (*E*_a_) was determined for ΔSL or sliding velocities (*V*) by using the Arrhenius equation; i.e., ΔSL or *V* = *V*_0_ exp (−*E*_a_/*RT*), where *V*_0_*, R,* and *T* are pre-exponential factor, gas constant (8.31 J/K/mol), and absolute temperature, respectively. The temperature coefficient *Q*_10_ (i.e., index of temperature dependence) was calculated from *E*_a_, minimal temperature (*T*_min_), and maximal temperature (*T*_max_):

$$Q_{10}={\left\{\exp\left[\frac{E_{a}}{R}\left(\frac{1}{T_{\min}}-\frac{1}{T_{\max}}\right)\right]\right\}}^{\frac{10}{T_{\max}-T_{\min}}}\text{.}$$

The error of *Q*_10_ (*ε*_*Q*_) was calculated from the fitting error of *E*_a_ (*ε*_*E*_) and the partial derivative of *Q*_10_ with respect to *E*_a_:

$$\varepsilon_{Q}=\sqrt{\varepsilon_{E}^{2}{\left(\frac{\partial Q_{10}}{\partial E_{a}}\right)}^{2}.}$$

$$\frac{\partial Q_{10}}{\partial E_{a}}=\frac{10}{RT_{\max}T_{\min}}*\exp\left\{\frac{10E_{a}}{RT_{\max}T_{\min}}\right\}\text{.}$$

Throughout the present study, data were expressed as the mean ± standard error of the mean (SEM). The only exception was the experiment on the SL fluctuation analysis with isolated single fast skeletal myofibrils; data were expressed as the mean ± SD. For multiple comparisons, Dunnett’s test was used. Statistical significance was assumed to be P < 0.05. NS indicates P > 0.05. *n* indicates the number of samples.

### Online supplemental material

**Figure S2.**
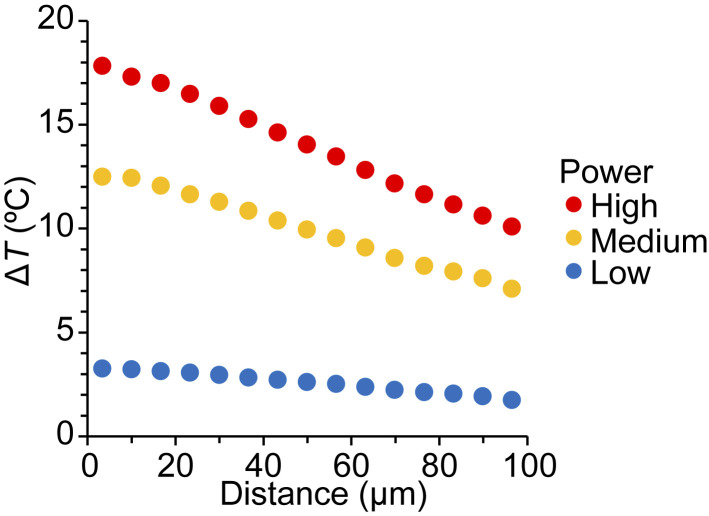
**Temperature gradients in an in vitro motility assay.** Changes in temperature (Δ*T*) generated by IR-laser irradiation were measured based on the changes in fluorescence intensity of rhodamine-labeled actin filaments. Three levels of gradients were adjusted by using neutral density filters: 60%, 50%, and 15% for high, medium, and low powers, respectively. Data are expressed as mean ± SEM. Error bars, <0.2°C (within plots). *n* = 3 for all power levels.

**Figure S3.**
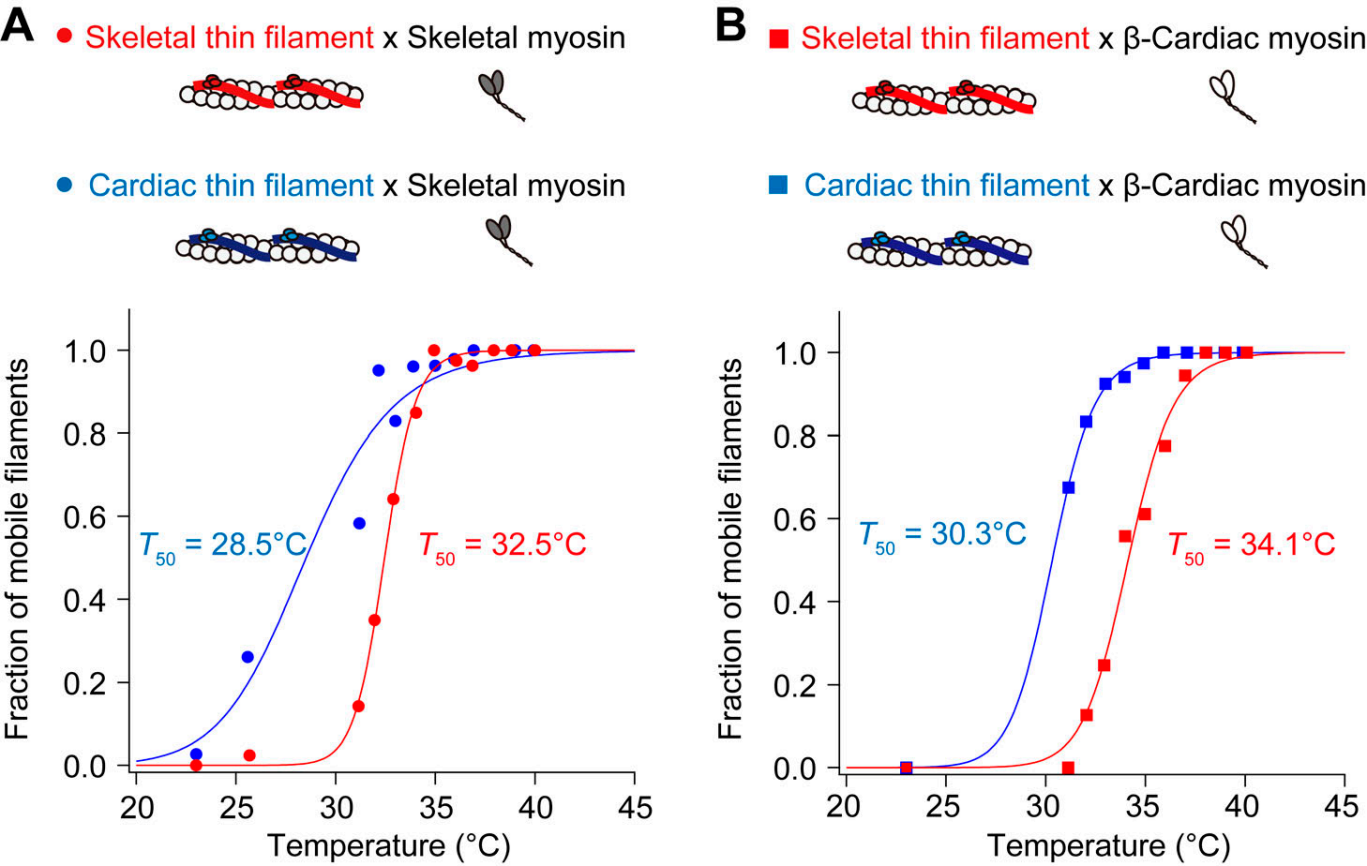
**Fraction of mobile filaments upon IR laser irradiation at pCa 9. (A)** Relationship between temperature and the fraction of mobile skeletal thin filaments (red closed circles) or cardiac thin filaments (blue closed circles) on skeletal myosin. Regression analysis was performed based on the Hill equation (see Materials and methods). The *T*_50_ values were 32.5°C and 28.5°C for skeletal and cardiac thin filaments, respectively. **(B)** Relationship between temperature and the fraction of mobile skeletal thin filaments (red closed squares) or cardiac thin filaments (blue closed squares) on β-cardiac myosin. The *T*_50_ values were 34.1°C and 30.3°C for skeletal and cardiac thin filaments, respectively. See Table S7 for details.

Fig. S1 shows the fluctuation analysis for the average length of five sarcomeres along a fast skeletal myofibril at rest. Fig. S2 shows temperature gradients in an in vitro motility assay. Fig. S3 shows the fraction of mobile filaments upon IR laser irradiation at pCa 9. Table S1 summarizes the sliding velocities obtained in the present in vitro motility assay experiments on skeletal myosin at pCa 9. Table S2 summarizes the sliding velocities obtained in the present in vitro motility assay experiments on skeletal myosin at pCa 5. Table S3 summarizes the sliding velocity ratios at pCa 9/pCa 5 in the present in vitro motility assay experiments on skeletal myosin. Table S4 summarizes the sliding velocities obtained in the present in vitro motility assay experiments on β-cardiac myosin at pCa 9. Table S5 summarizes the sliding velocities obtained in the present in vitro motility assay experiments on β-cardiac myosin at pCa 5. Table S6 summarizes the sliding velocity ratios at pCa 9/pCa 5 in the present in vitro motility assay experiments on β-cardiac myosin. Table S7 summarizes the fractions of mobile filaments in the present in vitro motility assay experiments at pCa 9. Table S8 summarizes the values of the temperature dependence obtained in our previous in vitro motility assay experiments (based on Ishii et al., 2019a). Video 1 shows sarcomere shortening induced by optical heating along a skeletal myofibril in relaxing solution. Videos 2, 3, and 4 show the thermal activation of reconstituted skeletal thin filaments on skeletal myosin at pCa 9 at the high, medium, and low heating levels, respectively. Video 5 shows thermal activation of reconstituted skeletal thin filaments on skeletal myosin at pCa 5. Video 6 shows thermal activation of reconstituted cardiac thin filaments on skeletal myosin at pCa 9. Video 7 shows thermal activation of reconstituted skeletal thin filaments on β-cardiac myosin at pCa 9. Video 8 shows thermal activation of reconstituted cardiac thin filaments on β-cardiac myosin at pCa 9.

## Results

**Figure 1.**
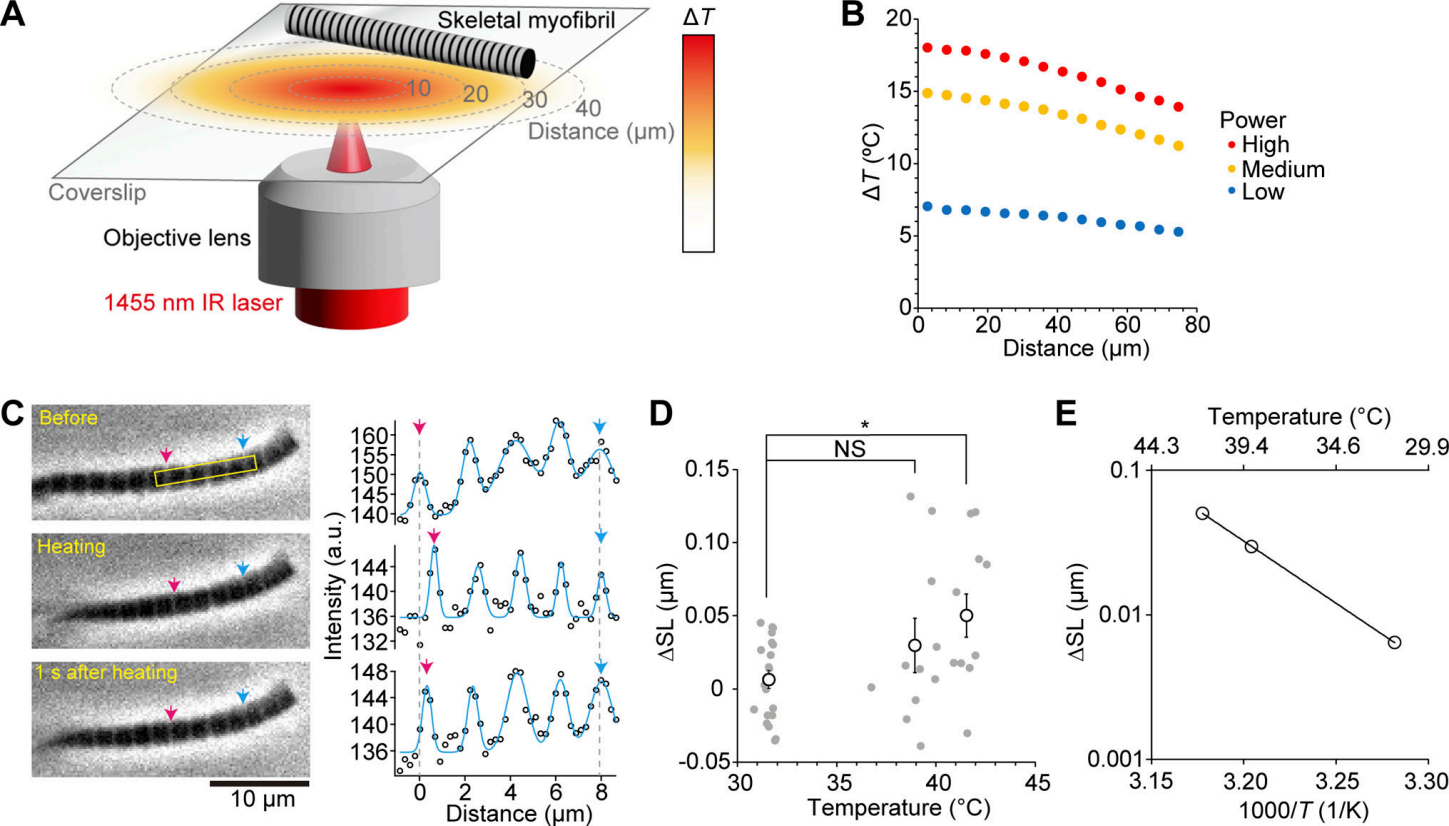
**Thermal activation of fast skeletal myofibrils immersed in relaxing solution. (A)** Schematic illustration of the experimental system. A rabbit psoas myofibril was immersed in relaxing solution on a coverslip. Temperature was directly increased by an IR laser beam (λ = 1,455 nm), which elicits sarcomere shortening as a function of the distance from the heat source (as shown by the red-yellow gradient; see the color bar on the right). **(B)** Changes in temperature (Δ*T*) generated by IR-laser irradiation. Three levels of gradients were adjusted by neutral density filters: 40%, 25%, and 15% for high, medium, and low powers, respectively. Data expressed as mean ± SEM. Error bars are <0.2°C (within plots). *n* = 3 for all power levels. **(C)** Left: Phase-contrast images of a skeletal myofibril in the relaxing solution (pCa 9). Top, middle, and bottom show images before, during, and 1 s after heating for 2 s, respectively. Sarcomeres within the yellow-outlined rectangle were analyzed. Pink and blue arrows in each image indicate the positions of Z-disks along a myofibril analyzed. Imaging was performed at 30 fps. Right: Intensity profiles of four sarcomeres along a myofibril shown left. Starting point on the x-axis (i.e., 0 µm) indicates the position of the Z-disk shown by a pink arrow before heating. The heat source was ∼28 µm away from the myofibril. Δ*T* was 17.1°C (cf. temperature gradient for high power in B). **(D)** Relationship of the temperature during heating versus changes in sarcomere length (ΔSL). ΔSL was calculated as SL before heating minus SL during heating. Each plot in the graph indicates the average shortening of sequentially connected four to six sarcomeres along a myofibril. *n* = 20, 10, and 11 myofibrils at 31.6 ± 0.1°C, 38.9 ± 0.3°C, and 41.5 ± 0.2°C, respectively. Data expressed as mean ± SEM. P is determined by Dunnett’s multiple comparison test. *P < 0.05. NS, not statistically significant. **(E)** Arrhenius plot for ΔSL. ΔSL is expressed in logarithm. Average values from D were used. ΔSL = 1.08 × 10^26^ exp (−1.65 × 10^5^/*RT*) (r^2^ = 1.00). *T*, absolute temperature. *R*, gas constant. r^2^, coefficient of determination. Experiments were performed at 25 ± 1°C.

First, we investigated whether and how microscopic heating induces sarcomere shortening along isolated fast skeletal myofibrils in a relaxing solution (pCa 9; as illustrated in Fig. 1 A). The magnitude of the temperature increase (Δ*T*) was 18°C, 15°C, and 7°C, respectively, by high, medium, and low powers of IR laser at the point where the laser was focused (0 μm in Fig. 1 B). Δ*T* decreased as a function of the distance from the irradiation point. We found that heating induced reversible shortening of sarcomeres; namely, sarcomeres were shortened upon laser irradiation, which persisted for 2 s during irradiation and then lengthened following cessation of irradiation (Fig. 1 C and Video 1). The relationship of temperature versus ΔSL obtained from 41 myofibrils (consisting of five to six sarcomeres), as summarized in Fig. 1 D, indicates that some myofibrils contracted at ∼40°C, and the significant SL shortening of 0.05 ± 0.01 μm (i.e., 2.2 ± 0.7%) was observed at 41.5 ± 0.2°C. It should be noted that this magnitude is comparable to the IR laser-induced shortening of skinned rat cardiomyocytes when irradiated for 0.5 s from 36 to ∼43°C (shortening, 2.0 ± 0.7%; Oyama et al., 2012). The Arrhenius plot analysis of ΔSL revealed that the activation energy *E*_a_ was 165 kJ/mol and the temperature dependence *Q*_10_ was 7.9 (Fig. 1 E).

**Video 1.** **Sarcomere shortening induced by optical heating along a skeletal myofibril in relaxing solution.** Phase-contrast image sequence of a fast skeletal myofibril in relaxing solution (pCa 9; see Fig. 1 C). IR laser beam was focused on the yellow circle at the high heating level (cf. Fig. 1 B). Δ*T* at the center of a myofibril, 17.1°C. Temperature before heating, 25°C. Scale bar, 10 μm. Imaging performed at 30 fps.

**Figure 2.**
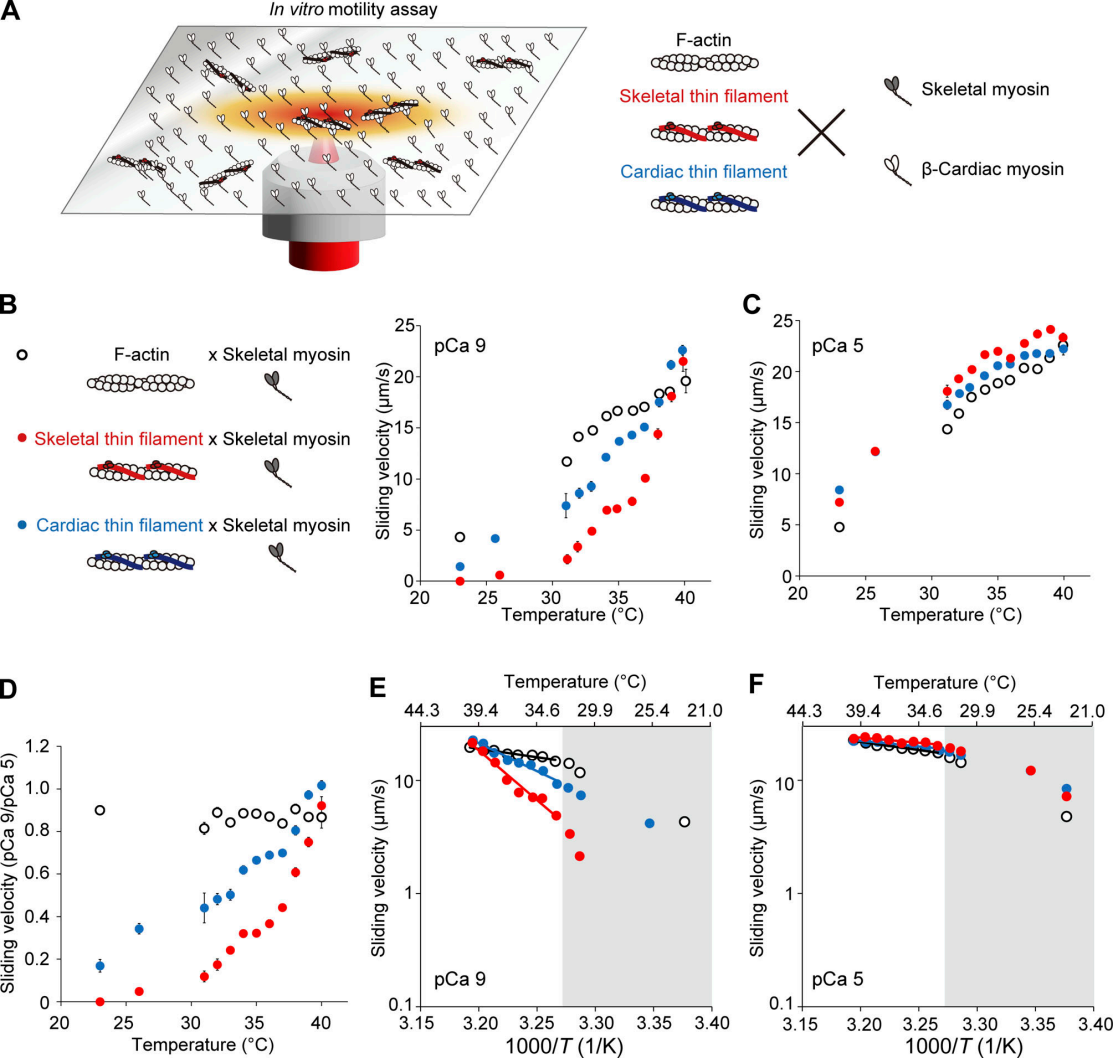
**Thermal activation of F-actin, fast skeletal thin filaments, and cardiac thin filaments on fast skeletal myosin in an in vitro motility assay. (A)** Schematic illustration showing the experimental system. Actin filaments (F-actin) or reconstituted skeletal or cardiac thin filaments interacted with skeletal or β-cardiac myosin treated on the glass coverslip in the absence (pCa 9) or presence (pCa 5) of Ca^2+^ (as shown on right). Temperature was increased by IR laser irradiation (from the baseline temperature of 23°C up to 40°C), inducing filament movements in various directions. The periods of heating were 2 and 10 s for skeletal and β-cardiac myosin, respectively. **(B)** Relationship between temperature and the sliding velocity of F-actin (black open circles), skeletal thin filaments (red closed circles), or cardiac thin filaments (blue closed circles) on skeletal myosin at pCa 9. *n* = 786, 684, and 665 for F-actin, skeletal thin filaments, and cardiac thin filaments, respectively. Data expressed as mean ± SEM. **(C)** Same as in B at pCa 5. *n* = 1,453, 1,296, and 1,442 for F-actin, skeletal thin filaments, and cardiac thin filaments, respectively. **(D)** Graph comparing the sliding velocity ratios at pCa 9/pCa 5 for F-actin, skeletal thin filaments, and cardiac thin filaments over the range of temperature. Data expressed as mean ± SEM. **(E)** Arrhenius plot for the sliding velocity of F-actin, skeletal thin filaments, and cardiac thin filaments at pCa 9. *T*, absolute temperature. Average values from B were used. Sliding velocity (*V*) is expressed in logarithm. F-actin: *V* = 1.09 × 10^6^ exp (−2.85 × 10^4^/*RT*) (r^2^ = 0.95). Skeletal thin filaments: *V* = 2.30 × 10^29^ exp (−1.68 × 10^5^/*RT*) (r^2^ = 0.98). Cardiac thin filaments: *V* = 1.27 × 10^17^ exp (−9.43 × 10^4^/*RT*) (r^2^ = 0.97). *Q*_10_, 1.4, 8.2, and 3.3 for F-actin, skeletal thin filaments and cardiac thin filaments, respectively. **(F)** Same as in E for F-actin, skeletal thin filaments, and cardiac thin filaments at pCa 5. Average values from C were used. F-actin: *V* = 8.15 × 10^5^ exp (−2.73 × 10^4^/*RT*) (r^2^ = 0.97). Skeletal thin filaments: *V* = 2.11 × 10 exp (−1.76 × 10^4^/*RT*) (r^2^ = 0.80). Cardiac thin filaments: *V* = 3.86 × 10^4^exp (−1.94 × 10^4^/*RT*) (r^2^ = 0.90). *Q*_10_, 1.4, 1.2, and 1.3 for F-actin, skeletal thin filaments and cardiac thin filaments, respectively. Data obtained below 32°C (shown in the grey region) were not employed for the fitting in E and F. See Tables S1, S2, and S3 for details.

To investigate the molecular basis for the heating-induced SL shortening in myofibrils, we analyzed the effects of heating on the sliding movements of reconstituted thin filaments in an in vitro motility assay (experimental design illustrated in Fig. 2 A). The change in local temperature was measured by taking advantage of the thermal quenching of rhodamine-labeled actin filaments (see Fig. S2). First, we performed experiments in the absence of Ca^2+^ (pCa 9) with F-actin or fast skeletal thin filaments on fast skeletal myosin (Fig. 2 B, Table S1, and Videos 2, 3, and 4). F-actin slid at 4.3 ± 0.06 μm/s at a room temperature of 23°C, but skeletal thin filaments did not move at that temperature indicating that Tm–Tn effectively suppresses the sliding of reconstituted thin filaments (cf., Ishii et al., 2019a). Optical heating to >30°C induced sliding of skeletal thin filaments. The sliding velocity which increased for both F-actin and skeletal thin filaments with increasing temperature, was significantly faster for F-actin compared to skeletal thin filaments in the range of 31–38°C. F-actin and skeletal thin filaments exhibited similar (P > 0.05) sliding velocities at higher temperatures of 39°C and 40°C. Under the activating condition in the presence of Ca^2+^ (pCa 5), skeletal thin filaments slid at 7.2 ± 0.12 μm/s at 23°C, significantly faster than F-actin (4.8 ± 0.05 μm/s; Fig. 2 C, Table S2, and Video 5). The sliding velocity which increased for both F-actin and skeletal thin filaments upon a rise of temperature, was significantly faster for skeletal thin filaments in the range of 23–39°C, but the difference was insignificant at 40°C. The finding at 23–39°C supports the notion that in the presence of Ca^2+^, Tm–Tn accelerates the actomyosin interaction (for details: e.g., Kawai et al., 2000; Oguchi et al., 2011; Ishii et al., 2018). Fig. 2 D compares the ratio of sliding velocities at pCa 9/pCa 5 between F-actin and skeletal thin filaments. While the ratio was nearly constant (within the range between 0.81 ± 0.03 and 0.91 ± 0.02) over the range of temperature (23–40°C) for F-actin, it increased upon a rise of temperature for skeletal thin filaments, but it was significantly less at 31–39°C compared with F-actin. At a body temperature of 37°C, the value was 0.44 ± 0.01, suggesting that skeletal thin filaments are partially activated at the relaxed state under the physiological condition, a characteristic similar to that recently reported by us for cardiac thin filaments (Ishii et al., 2019a). It is therefore reasonable to consider that in both skeletal and cardiac muscles, the intrinsic partial activation at rest allows for rapid contraction in response to a rise of [Ca^2+^]_i_ during activation.

**Video 2.** **Thermal activation of reconstituted skeletal thin filaments on skeletal myosin at pCa 9 at the high heating level.** Fluorescence image sequence of reconstituted fast skeletal thin filaments on fast skeletal myosin in relaxing solution (pCa 9; see Fig. 2 B). IR laser beam was focused at the yellow circle at the high heating level (cf. Fig. S2). Δ*T* at 10, 30, and 50 μm away from the heat source, 17°C, 16°C, and 14°C, respectively. Temperature before heating, 23°C. Scale bar, 10 μm. Imaging performed at 30 fps.

**Video 3.** **Thermal activation of reconstituted skeletal thin filaments on skeletal myosin at pCa 9 at the medium heating level.** Fluorescence image sequence of reconstituted fast skeletal thin filaments on fast skeletal myosin in relaxing solution (pCa 9; see Fig. 2 B). IR laser beam was focused at the yellow circle at the medium heating level (cf. Fig. S2). Δ*T* at 10, 30, and 50 μm away from the heat source, 12°C, 11°C, and 10°C, respectively. Temperature before heating, 23°C. Scale bar, 10 μm. Imaging performed at 30 fps.

**Video 4.** **Thermal activation of reconstituted skeletal thin filaments on skeletal myosin at pCa 9 at the low heating level.** Fluorescence image sequence of reconstituted fast skeletal thin filaments on fast skeletal myosin in relaxing solution (pCa 9; see Fig. 2 B). IR laser beam was focused at the yellow circle at the low heating level (cf. Fig. S2). Δ*T* at 10, 30, and 50 μm away from the heat source, 3.2°C, 3.0°C, and 2.6°C, respectively. Temperature before heating, 23°C. Scale bar, 10 μm. Imaging performed at 30 fps.

**Video 5.** **Thermal activation of reconstituted skeletal thin filaments on skeletal myosin at pCa 5.** Fluorescence image sequence of reconstituted fast skeletal thin filaments on fast skeletal myosin in activating solution (pCa 5; see Fig. 2 C). IR laser beam was focused at the yellow circle at the high heating level (cf. Fig. S2). Δ*T* at 10, 30, and 50 μm away from the heat source, 17°C, 16°C, and 14°C, respectively. Temperature before heating, 23°C. Scale bar, 10 μm. Imaging performed at 30 fps.

We then investigated the sliding movements of reconstituted cardiac thin filaments on fast skeletal myosin and compared the data with those obtained with fast skeletal thin filaments. At pCa 9, cardiac thin filaments moved slowly at 23°C (1.4 ± 0.25 μm/s) and 26°C (4.2 ± 0.30 μm/s; Fig. 2 B and Video 6). The sliding velocity was significantly faster for cardiac thin filaments at 31–39°C and became similar to that of skeletal thin filaments at 40°C. At pCa 5, the sliding velocity was significantly faster for cardiac thin filaments at 23°C; however, it was slightly but significantly faster for skeletal thin filaments than cardiac thin filaments at higher temperatures (i.e., 32–35°C and 37–39°C) and became similar between skeletal and cardiac thin filaments at 40°C (Fig. 2 C). Comparison of the ratios of sliding velocities at pCa 9/pCa 5 revealed that the values were higher for cardiac thin filaments than skeletal thin filaments, with the differences significant at 31–39°C (Fig. 2 D and Table S3). It can therefore be said that cardiac thin filaments are apparently more heat-sensitive than skeletal thin filaments. This finding suggests that Ca^2+^-dependent activation of skeletal thin filaments is suppressed to a greater extent by Tm–Tn compared with cardiac thin filaments over the wide range of temperature, including the body temperature range, i.e., from 31°C to 39°C.

**Video 6.** **Thermal activation of reconstituted cardiac thin filaments on skeletal myosin at pCa 9.** Fluorescence image sequence of reconstituted cardiac thin filaments on fast skeletal myosin in relaxing solution (pCa 9; see Fig. 2 B). IR laser beam was focused at the yellow circle at the high heating level (cf. Fig. S2). Δ*T* at 10, 30, and 50 μm away from the heat source, 17°C, 16°C, and 14°C, respectively. Temperature before heating, 23°C. Scale bar, 10 μm. Imaging performed at 30 fps.

To understand the temperature dependence, we analyzed the data of F-actin and skeletal thin filaments by using Arrhenius plots. We found that the temperature dependence was higher for skeletal thin filaments than F-actin; the *Q*_10_ values were 1.4 and 8.2 for F-actin and skeletal thin filaments, respectively, at pCa 9 (Fig. 2 E and Table 1), indicating that Tm–Tn increases the temperature dependence of the actomyosin interaction ∼5.9-fold. It is noteworthy that *Q*_10_ for skeletal thin filaments was comparable to that obtained in the myofibrillar shortening experiment (i.e., 7.9; Fig. 1 E). At pCa 5, the *Q*_10_ values were similar to those obtained for F-actin at pCa 9, i.e., 1.4 and 1.2 for F-actin and skeletal thin filaments, respectively (Fig. 2 F).

**Table 1.** Summary of the values of the temperature dependence obtained in the present in vitro motility assay experiments

Myosin type	Thin filament type	pCa	*Q*_10_	*E*_a_ (kJ/mol)
Skeletal	F-actin	9	1.4 ± 0.05	28.5 ± 2.8
		5	1.4 ± 0.03	27.3 ± 1.9
	Skeletal	9	8.2 ± 1.3	168 ± 13
		5	1.2 ± 0.06	17.6 ± 3.6
	Cardiac	9	3.3 ± 0.3	94.3 ± 7.3
		5	1.3 ± 0.04	19.4 ± 2.7
β-cardiac	F-actin	9	3.5 ± 0.3	100 ± 7.7
		5	3.1 ± 0.2	90.9 ± 4.6
	Skeletal	9	30 ± 16	272 ± 41
		5	2.6 ± 0.1	76.6 ± 4.3
	Cardiac	9	5.1 ± 1.2	130 ± 19
		5	1.5 ± 0.09	33.7 ± 4.6

The values of *Q*_10_ and *E*_a_ were determined from the sliding velocities between 33°C and 40°C (see Materials and methods). Data expressed as mean ± SEM.

Regarding the temperature dependence of skeletal versus cardiac thin filaments at pCa 9, *Q*_10_ was 3.3 for cardiac thin filaments, as compared with 8.2 for skeletal thin filaments (Fig. 2 E and Table 1). It is therefore reasonable to consider that the temperature dependence is ∼2.5 times higher for skeletal thin filaments than cardiac thin filaments when interacting with skeletal myosin in an in vitro motility assay. At pCa 5, the values were similar with 1.2 and 1.3 for skeletal and cardiac thin filaments, respectively (Fig. 2 F).

**Figure 3.**
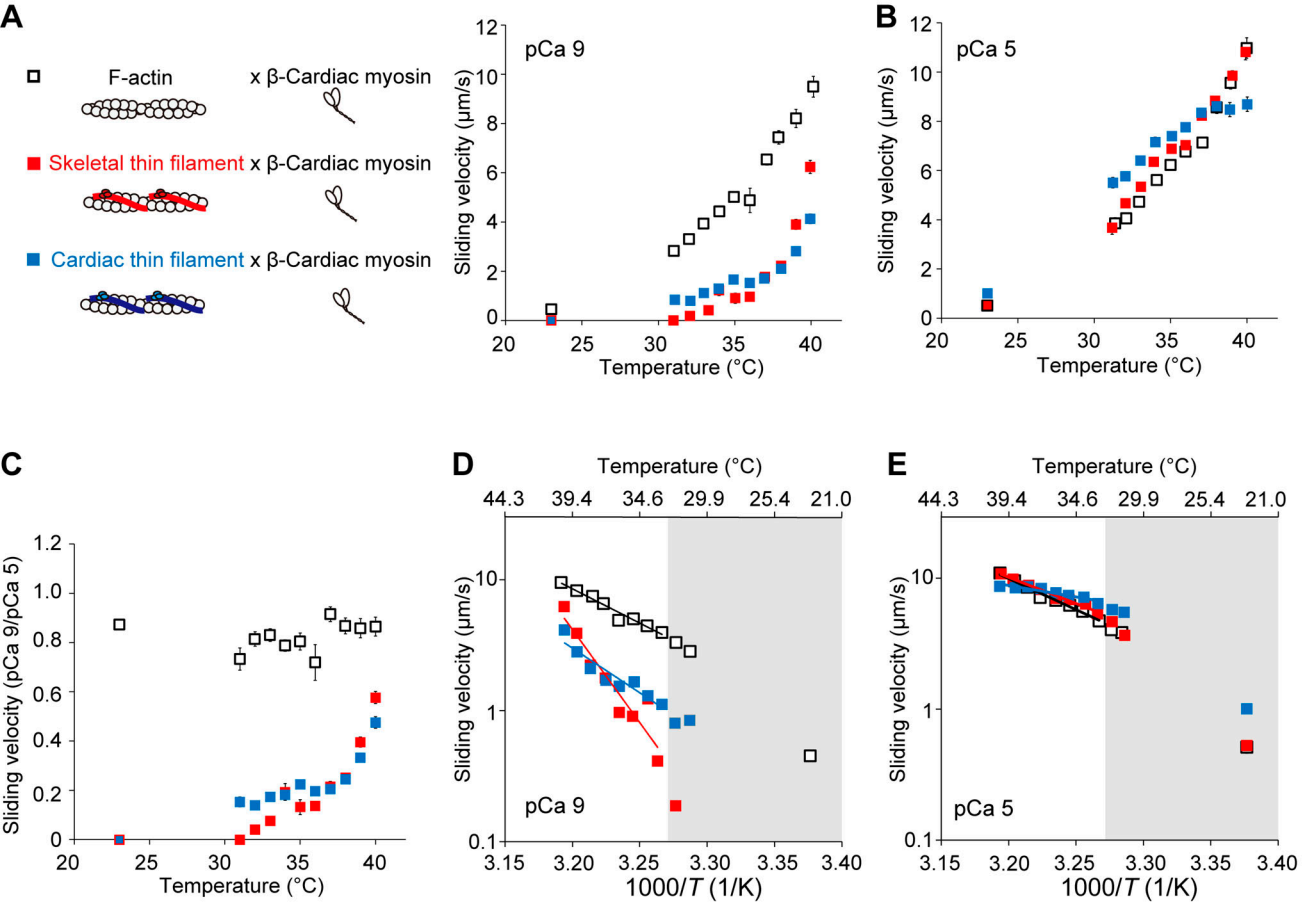
**Thermal activation of F-actin, fast skeletal thin filaments, and cardiac thin filaments on β-cardiac myosin in an in vitro motility assay. (A)** Relationship between temperature and the sliding velocity of F-actin (black open squares), skeletal thin filaments (red closed squares), and cardiac thin filaments (blue closed squares) on β-cardiac myosin at pCa 9. Temperature was increased from the baseline of 23°C by IR-laser irradiation for 10 s. *n* = 545, 354, and 720 for F-actin, skeletal thin filaments, and cardiac thin filaments, respectively. Data expressed as mean ± SEM. **(B)** Same as in A at pCa 5. *n* = 931, 1,121, and 872 for F-actin, skeletal thin filaments, and cardiac thin filaments, respectively. **(C)** Graph comparing the sliding velocity ratios at pCa 9/pCa 5 for F-actin and skeletal thin filament over the range of temperature. Data expressed as mean ± SEM. **(D)** Arrhenius plot for the sliding velocity of F-actin, skeletal thin filaments, and cardiac thin filaments at pCa 9. *T*, absolute temperature. Average values from A were used. Sliding velocity (*V*) is expressed in logarithm. F-actin: *V* = 5.25 × 10^17^ exp (−1.00 × 10^5^/*RT*) (r^2^ = 0.98). Skeletal thin filaments: *V* = 1.43 × 10^46^ exp (−2.72 × 10^5^/*RT*) (r^2^ = 0.95). Cardiac thin filaments: *V* = 2.00 × 10^22^ exp (−1.30 × 10^5^/*RT*) (r^2^ = 0.88). *Q*_10_, 3.5, 30, and 5.1 for F-actin, skeletal thin filaments, and cardiac thin filaments, respectively. **(E)** Same as in D for F-actin, skeletal thin filaments, and cardiac thin filaments at pCa 5. Average values from B were used. F-actin: *V* = 1.57 × 10^16^ exp (−9.09 × 10^4^/*RT*) (r^2^ = 0.99). Skeletal thin filaments: *V* = 6.57 × 10^13^ exp (−7.66 × 10^4^/*RT*) (r^2^ = 0.99). Cardiac thin filaments: *V* = 3.76 × 10^6^ exp (−3.37 × 10^4^/*RT*) (r^2^ = 0.89). *Q*_10_, 3.1, 2.6, and 1.5 for F-actin, skeletal thin filaments, and cardiac thin filaments, respectively. Data obtained below 32°C (shown in the gray region) were not employed for the fitting in D and E. See Tables S4, S5, and S6 for details.

It is well known that the sliding velocity of F-actin in an in vitro motility assay is slower on β-cardiac myosin than on fast skeletal myosin (e.g., Ishii et al., 2019a and references therein). Therefore, we investigated the sliding movements of F-actin and fast skeletal thin filaments on β-cardiac myosin. At 23°C, F-actin slid at 0.4 ± 0.01 μm/s, but skeletal thin filaments did not move at pCa 9 (Fig. 3 A, Table S4, and Video 7; as on skeletal myosin; cf. Fig. 2 B). Optical heating induced the sliding of skeletal thin filaments, and the sliding velocity increased for both F-actin and skeletal thin filaments with increasing temperature; it was significantly faster for F-actin at 32–40°C. Unlike the case of sliding on skeletal myosin (cf. Fig. 2 C), F-actin slid significantly faster than skeletal thin filaments even at the highest temperature of 40°C. At pCa 5, skeletal thin filaments slightly but significantly slid faster than F-actin at 32–35°C and 37°C; however, the sliding velocity was similar between the two types of filaments at 38–40°C (Fig. 3 B and Table S5). Comparison of the ratio of sliding velocities at pCa 9/pCa 5 revealed that it was nearly constant (within the range between 0.72 ± 0.07 and 0.92 ± 0.03) for F-actin; it increased with increasing temperature for skeletal thin filaments, but significantly less than F-actin at 33–40°C (Fig. 3 C and Table S6). At 37°C, the ratio for skeletal thin filaments was 0.21 ± 0.02, clearly lower than the value obtained with skeletal myosin (i.e., 0.44 ± 0.01 in Fig. 2 D). These results suggest that skeletal myosin partially activates skeletal thin filaments at/around body temperature in a more effective fashion compared with β-cardiac myosin, consistent with our previous finding with cardiac thin filaments (cf. Ishii et al., 2019a).

**Video 7.** **Thermal activation of reconstituted skeletal thin filaments on β-cardiac myosin at pCa 9.** Fluorescence image sequence of reconstituted fast skeletal thin filaments on β-cardiac myosin in relaxing solution (pCa 9; see Fig. 3 A). IR laser beam was focused at the yellow circle at the high heating level (cf. Fig. S2). Δ*T* at 10, 30, and 50 μm away from the heat source, 17°C, 16°C, and 14°C, respectively. Temperature before heating, 23°C. Scale bar, 10 μm. Imaging performed at 30 fps.

We then investigated the sliding movements of cardiac thin filaments on β-cardiac myosin and compared the data with those obtained with fast skeletal thin filaments. At pCa 9, cardiac thin filaments did not move at 23°C, as was the case for skeletal thin filaments (Fig. 3 A); they started to slide at 31°C (Fig. 3 A and Video 8). Cardiac thin filaments slid significantly faster than skeletal thin filaments at 32°C, 33°C, 35°C, and 36°C (i.e., lower than at body temperature); however, at high temperatures of 39°C and 40°C, skeletal thin filaments slid faster than cardiac thin filaments (6.2 ± 0.26 and 4.1 ± 0.20 μm/s at 40°C for skeletal and cardiac thin filaments, respectively). At pCa 5, cardiac thin filaments slid significantly faster than skeletal thin filaments below body temperature, i.e., at 23–36°C (Fig. 3 B). At 39 and 40°C, skeletal thin filaments slid significantly faster than cardiac thin filaments (10.8 ± 0.32 and 8.7 ± 0.30 μm/s at 40°C for skeletal and cardiac thin filaments, respectively). Fig. 3 C compares the ratio of sliding velocities at pCa 9/pCa 5 between skeletal and cardiac thin filaments. We found a gap at/around body temperature; namely, while the ratio was significantly higher for cardiac thin filaments at 35°C and 36°C, it was significantly higher for skeletal thin filaments at 39°C and 40°C.

**Video 8.** **Thermal activation of reconstituted cardiac thin filaments on β-cardiac myosin at pCa 9.** Fluorescence image sequence of reconstituted cardiac thin filaments on β-cardiac myosin in relaxing solution (pCa 9; see Fig. 3 A). IR laser beam was focused at the yellow circle at the high heating level (cf. Fig. S2). Δ*T* at 10, 30, and 50 μm away from the heat source, 17°C, 16°C, and 14°C, respectively. Temperature before heating, 23°C. Scale bar, 10 μm. Imaging performed at 30 fps.

Arrhenius plots revealed that *Q*_10_ for F-actin on β-cardiac myosin was 3.5 (Fig. 3 D and Table 1), i.e., ∼2.5-fold higher than that obtained with skeletal myosin at pCa 9 (cf. Fig. 2 E: *Q*_10_ 1.4). Surprisingly, *Q*_10_ was 30 for skeletal thin filaments, i.e., ∼3.7-fold higher than that obtained with skeletal myosin (cf. Fig. 2 E: *Q*_10_ 8.2). It can therefore be said that the temperature dependence is higher for β-cardiac myosin than skeletal myosin, and the difference becomes pronounced with Tm–Tn. As with skeletal myosin (Fig. 2 F), the *Q*_10_ values were similar with 3.1 and 2.6 for F-actin and skeletal thin filaments, respectively, at pCa 5 (Fig. 3 E and Table 1). This finding strengthens the assumption that in the presence of Ca^2+^, the temperature dependence of skeletal thin filaments becomes similar to that of F-actin.

*Q*_10_ at pCa 9 was 5.1 for cardiac thin filaments as compared to 30 for skeletal thin filaments (Fig. 3 D and Table 1). It can therefore be said that the greater temperature dependence of skeletal thin filaments, as compared to cardiac thin filaments, on skeletal myosin (*Q*_10_: 8.2 versus 3.3; cf. Fig. 2 D), becomes more pronounced on β-cardiac myosin. At pCa 5, the *Q*_10_ values decreased to 2.6 and 1.5 for skeletal and cardiac thin filaments, respectively, smaller than those obtained for F-actin, i.e., 3.5 and 3.1 at pCa 9 and 5, respectively (Fig. 3, D and E; and Table 1).

Finally, we quantified the threshold temperature (*T*_50_) at which 50% of skeletal or cardiac thin filaments started to slide at pCa 9. *T*_50_ was determined from the fraction of mobile filaments (Fig. S3, A and B; and Table S7). We found that (a) *T*_50_ for cardiac thin filaments was 28.5°C on skeletal myosin, lower than that for skeletal thin filaments (32.5°C), and (b) *T*_50_ for cardiac thin filaments was 30.3°C on β-cardiac myosin, lower than that for skeletal thin filaments (34.1°C). These findings support the notion that cardiac thin filaments are apparently more heat-sensitive than skeletal thin filaments on either type of myosin.

## Discussion

In the present study, we demonstrated that sarcomeres were shortened by microheating along isolated fast skeletal myofibrils immersed in relaxing solution. The temperature dependence for sarcomere shortening was comparable to that obtained in an in vitro motility assay with reconstituted fast skeletal thin filaments on fast skeletal myosin at pCa 9, with the *Q*_10_ values 7.9 and 8.2 in myofibrils and in an in vitro motility assay, respectively. It is therefore reasonable to consider that the data obtained from an in vitro motility assay reflects the temperature dependence of myofibrillar contractile ability in the absence of Ca^2+^. The threshold temperature to induce significant sarcomere shortening and thin filament sliding differed by ∼10°C, i.e., ∼40°C and ∼30°C for the former and latter, respectively (Figs. 1 and 2). The higher threshold temperature for myofibrils may be due primarily to the partial attachment of myofibrils, especially their A-band surface, to the glass surface; namely, myofibrils are not under the complete unloaded condition. It is likewise possible that the myofilament lattice structure and the presence of accessory proteins, such as myosin-binding protein C in myofibrils, contribute to raising the threshold temperature. Therefore, heating-induced changes in SL will be decreased due to the resultant friction and the threshold temperature will accordingly be increased. We conclude that an in vitro motility assay combined with optical heating is more desirable in analyzing the in-depth nature of the temperature dependence of contractile function in striated muscle sarcomeres at/around body temperature. We discuss the present findings based on thermal regulation by myosin and thin filament regulatory proteins.

It has been reported that unloaded sliding velocity in an in vitro motility assay is determined by ATPase rate, step size, duty ratio, and the number of force-generating cross-bridges (e.g., Uyeda et al., 1990; Ishii et al., 2019b). Walklate et al. (2016) pointed out that skeletal (type IIX) myosin exhibits an ATPase rate 10-fold higher than that of β-cardiac myosin, but step size and duty ratio are similar (<2-fold difference) between the two types of myosin. Accordingly, we discuss the present findings on fast skeletal versus β-cardiac myosin, with an emphasis on the difference in the ATPase rate.

In the present study, the sliding velocity of F-actin monotonically increased upon a rise in temperature, i.e., ∼20 and ∼10 μm/s at 40°C on skeletal and β-cardiac myosin, respectively (Figs. 2 and 3). Unlike reconstituted thin filaments that did not slide at the baseline temperature of 23°C at pCa 9 on either type of myosin (Figs. 2 and 3), F-actin, albeit slowly, slid at 23°C; i.e., at ∼4.5 and ∼0.5 μm/s on skeletal and β-cardiac myosin, respectively, regardless of the presence or absence of Ca^2+^. It can therefore be said that under the present experimental condition, (a) Tm–Tn effectively suppresses actomyosin interaction and (b) the heating-induced increase in the velocity of F-actin primarily reflects the acceleration of the actomyosin ATPase activity. It is indeed widely regarded that myosin ATPase is increased with increasing temperature in the absence and presence of F-actin (Bárány, 1967). Earlier, Siemankowski et al. (1985) showed that the ADP-release rate at the end of the crossbridge cycling is rate-limiting in unloaded shortening of various types of muscles from rabbits (i.e., fast skeletal, slow skeletal, and cardiac muscles). Nyitrai et al. (2006) concluded that the ADP release is the rate-limiting step for the sliding of F-actin on HMM at temperatures between room temperature (∼25°C) and body temperature. Later, Yengo et al. (2012) compared in vitro motility assay experiments and stopped-flow measurements of ADP-release with HMM of chicken myosin V, gizzard smooth muscle, and mouse non-muscle myosin IIA. Their results demonstrated that the rate-limiting ADP-release step is accelerated in response to an increase in temperature with a similar temperature dependency of velocity in an in vitro motility assay. In the present study, we found that at 37°C, the sliding velocities of F-actin were 17 ± 0.34 and 6.5 ± 0.21 μm/s on skeletal and β-cardiac myosin, respectively, at pCa 9 (20 ± 0.36 and 7.1 ± 0.19 μm/s on skeletal and β-cardiac myosin, respectively, at pCa 5; Figs. 2 and 3). It is therefore likely that the ADP-release rate during unloaded shortening is approximately three times higher for fast skeletal myosin than cardiac β-myosin at physiological body temperature.

**Figure 4.**
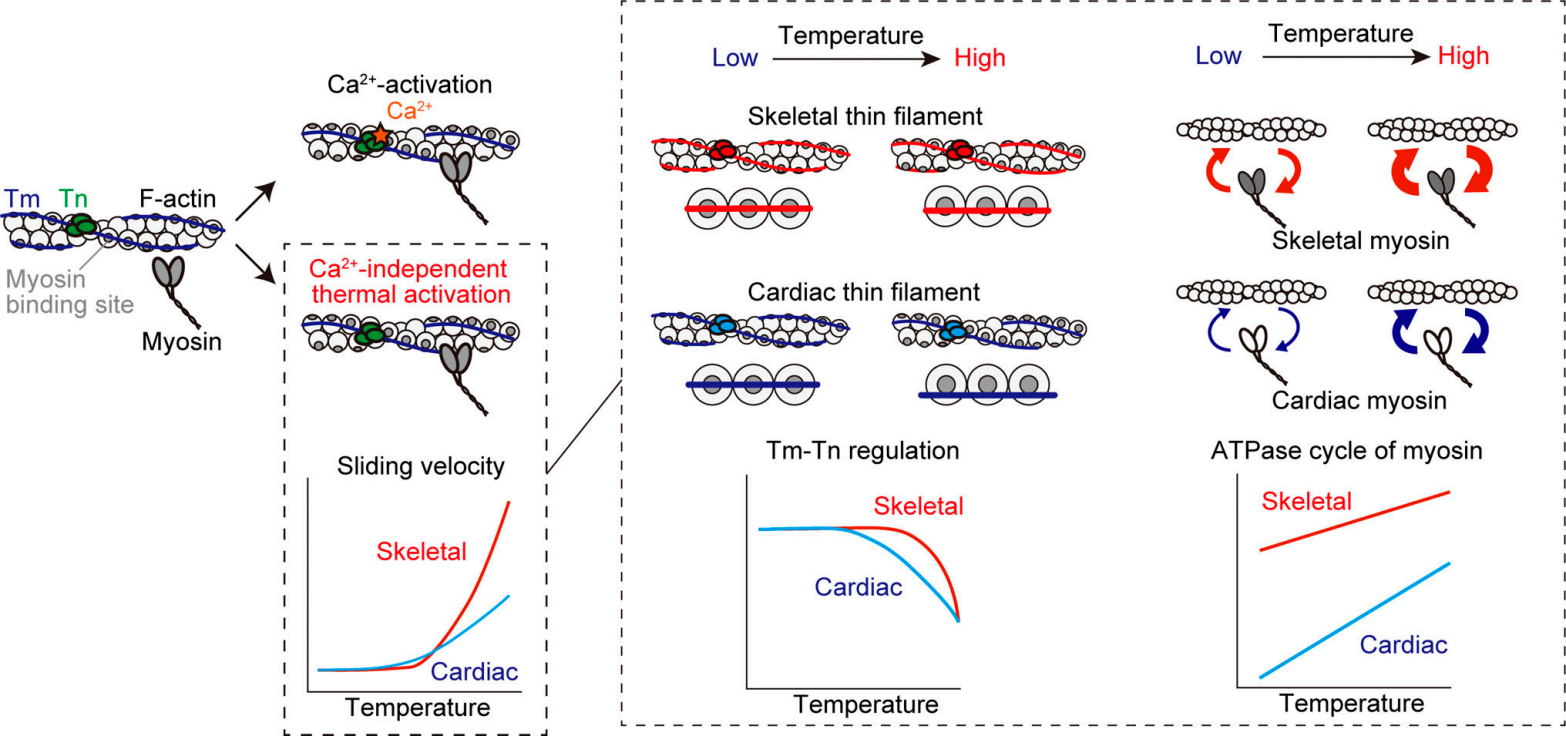
**Proposed complementary effects of myosin and regulatory proteins on the temperature dependence of sarcomeric activation in fast skeletal versus cardiac muscle.** Left: Regulation of the thin filament state by Ca^2+^ (top) or heating (bottom). Ca^2+^ binding to TnC shifts the thin filament state from off to on (top). Heating induces Ca^2+^-independent thermal activation of thin filaments via partial dissociation of Tm–Tn from F-actin (or weakening of the binding of Tm–Tn to F-actin), resulting in sliding movements of thin filaments in an in vitro motility assay (sliding velocity faster with fast skeletal Tm–Tn on fast skeletal myosin [red line] than with cardiac Tm–Tn on cardiac myosin [blue line]; see Figs. 2 and 3). Right: Complementary relationship between temperature and Tm–Tn regulation (left) or ATPase cycle of myosin (right) that causes Ca^2+^-independent thermal activation observed in the present study. Curved arrows indicate the rates of attachment or detachment of myosin to actin. The present as well as previous studies (Chandy et al., 1999; Houmeida et al., 2010; Risi et al., 2017; Heeley et al., 2002, 2006, 2019) suggest that the inhibitory effect of Tm–Tn on the thin filament state (i.e., Tm–Tn regulation) against a rise of temperature is weaker in cardiac muscle (blue line) than in fast skeletal muscle (red line). Therefore, an increase in temperature to within the body temperature range activates thin filaments to a greater magnitude in fast skeletal muscle (high *Q*_10_) than in cardiac muscle (low *Q*_10_). The ATPase cycle of fast skeletal myosin (red line) is faster than that of β-cardiac myosin (blue line) (Walklate et al., 2016), which results in a relative loss of recruitable heads in fast skeletal muscle at temperatures lower than body temperature. Accordingly, an increase in temperature to the body temperature range activates myosin to a greater magnitude in cardiac muscle (high *Q*_10_) than in fast skeletal muscle (low *Q*_10_). This way, the temperature dependence of striated muscle contraction is regulated by Tm–Tn and myosin in a complemental manner.

When interacting with F-actin, skeletal myosin exhibited lower temperature dependence (lower *Q*_10_) compared with cardiac β-myosin (Figs. 2 and 3). Indeed, the *Q*_10_ values for skeletal versus β-cardiac myosin with F-actin were 1.4 versus 3.5 and 1.4 versus 3.1 at pCa 9 and 5, respectively (Table 1). It is to be noted that the present *Q*_10_ values were similar to those obtained in previous studies; i.e., ∼1.9 calculated from the activation energy of 50 kJ/mol between 30°C and 45°C (Kato et al., 1999) for rabbit fast skeletal myosin and ∼3.1 for rabbit cardiac myosin between 19°C and 29°C (Yamashita et al., 1994). We consider that on fast skeletal myosin, a rapid release of ADP from the actomyosin complex allows for the prompt binding of ATP and the subsequent formation of working crossbridges, even at temperatures lower than body temperature (e.g., <30°C). This property leads to a decrease in the fraction of “recruitable” S1 heads needed for activation upon IR-laser irradiation (small *Q*_10_). Conversely, a higher fraction of recruitable S1 heads will be present on β-cardiac myosin in the same temperature range due to a comparatively slow ADP release from the actomyosin complex (large *Q*_10_; Fig. 4).

It is well known that myosin molecules in striated muscle sarcomeres can be in a biochemical state with an extremely low ATP turnover rate; i.e., the super-relaxed state (SRX; e.g., Stewart et al., 2010; Cooke, 2011; Irving, 2017; Craig and Padrón, 2022). The SRX is in equilibrium with the other biochemical state, i.e., disordered-relaxed state (DRX). It is considered that the biochemical state differs from the structural state of myosin molecules; however, S1 heads in DRX may be structurally in closer proximity to thin filaments (e.g., Cooke, 2011; Fusi et al., 2015). Therefore, a decrease in the number of myosin molecules in SRX is likely to result in an increase in the number of S1 heads available to bind to actin (e.g., Schmid and Toepfer, 2021). It has been reported that the cooperative transition of myosin from SRX to the active state via DRX is higher in fast skeletal muscle (Stewart et al., 2010) than in cardiac muscle (Hooijman et al., 2011). The acceleration of the transition to the active state decreases the fraction of recruitable S1 heads; therefore, the cooperativity difference may in part account for the lower *Q*_10_ value for skeletal myosin compared with β-cardiac myosin when interacting with F-actin (i.e., 1.4 versus 3.5). It has likewise been reported that the fraction of myosin in SRX is increased as a function of temperature in the range of 12−30°C in rabbit fast skeletal fibers (Stewart et al., 2010); however, the fraction is stable for bovine ventricular HMM in solution at 20−35°C (Rohde et al., 2018). Future studies are needed to investigate whether and how changes in the biochemical SRX–DRX equilibrium, as well as the structural on–off state of myosin molecules, contribute to the differing temperature dependence of thermal activation in skeletal versus cardiac muscle, especially within the body temperature range.

One may point out that the *Q*_10_ values obtained in the present study are smaller than those obtained in our previous study (Ishii et al., 2019a) for both fast skeletal and β-cardiac myosin when interacting with F-actin (at pCa 5 and 9) and cardiac thin filaments at pCa 5. Namely, the values in the present versus previous studies were 1.4 versus 2.4 (1.4 versus 2.6) and 3.5 versus 5.0 (3.1 versus 4.7) for skeletal and β-cardiac myosin with F-actin at pCa 9 (pCa 5), respectively, and 1.3 versus 1.9 and 1.5 versus 3.5 for skeletal and β-cardiac myosin with cardiac thin filaments at pCa 5, respectively. It is likely that these differences in *Q*_10_ are due to the differing temperature range; namely, we determined *Q*_10_ in the range between room temperature (24–25°C) and ∼41°C in the previous study, but between 33 and 40°C in the present study. Accordingly, we recalculated the values of *Q*_10_ and *E*_a_ in the range between 34 and 41°C for skeletal myosin and between 37 and 40°C for β-cardiac myosin based on the previously reported data (see Ishii et al., 2019a; Table S8). It is clearly seen that the recalculated *Q*_10_ and *E*_a_ values are closer to those obtained in the present study. Slightly lesser values for skeletal myosin in the present study may be due to a batch-to-batch difference because similar values were obtained for β-cardiac myosin in the previous and present studies.

Recently, we reported that the on–off equilibrium of the cardiac thin filament state is under the influence of ambient temperature, especially within the body temperature range; namely, an increase in temperature by IR laser irradiation shifts the equilibrium toward the on state, causing thin filament sliding movements in an in vitro motility assay (Ishii et al., 2019a). Earlier, Ishiwata (1978) demonstrated in solution that Tm–Tn dissociates from F-actin with increasing temperature (see Introduction). We consider that the heating-induced “partial dissociation” of Tm–Tn from F-actin (or weakening of the binding of Tm–Tn to F-actin; Fig. 4) accounts at least in part for the sliding movements of thin filaments at pCa 9 under all experimental conditions in the present study, i.e., either skeletal or cardiac thin filaments on skeletal or β-cardiac myosin. As evidenced by earlier studies on isolated rabbit skeletal myofibrils in that the activating effect of Ca^2+^ becomes less associated with an increase in temperature (Mühlrad and Hegyi, 1965; Murphy and Hasselbach, 1968), heating may have an effect apparently similar to that of Ca^2+^ in the activation of thin filaments. Here, it is to be noted that earlier studies analyzed the relationship between activation energy and actomyosin ATPase or myosin binding to thin filaments: first, Mühlrad and Hegyi (1965) demonstrated that the energy for myofibrillar ATPase becomes higher upon a decrease in the Ca^2+^ concentration. Second, Longyear et al. (2017) assumed from their single molecule findings that the energy required for the binding of myosin to thin filaments increases upon a decrease in the occupancy of Tn with Ca^2+^. We therefore consider that thermodynamic instability of Tm–Tn on F-actin is a physical factor that impacts the amplitude of the energy barrier for myosin binding to thin filaments and the ensuing actomyosin ATPase. Consequently, as discussed below, the apparent sensitivity to heating is changed under conditions with varying protein combinations in an in vitro motility assay.

We found that while apparent sensitivity to heating was higher for cardiac thin filaments than skeletal thin filaments on either skeletal (Fig. 2) or β-cardiac myosin (Fig. 3) at pCa 9, the *Q*_10_ values were greater for skeletal thin filaments than cardiac thin filaments, regardless of the type of myosin; i.e., 8.2 versus 3.3 on skeletal myosin and 30 versus 5.1 on β-cardiac myosin (difference, ∼2.5 and ∼5.9 times for the former and latter, respectively). In relation to the partial dissociation of Tm–Tn from F-actin, a later study by Chandy et al. (1999) revealed that the steady-state phosphorescence anisotropy values for rabbit skeletal and cardiac Tm were 0.025 ± 0.005 and 0.010 ± 0.003 at 20°C, respectively. Their analyses indicate that (a) cardiac Tm is more mobile than skeletal Tm, and (b) the increased mobility on the surface of F-actin reflects either the rotational motion of a smaller physical unit of Tm or the torsional twisting of the molecule. We consider that because of the thermodynamically instable nature of Tm–Tn on F-actin, cardiac thin filaments are more heat-sensitive than skeletal thin filaments at pCa 9, regardless of the type of myosin (cf., Figs. 2 and 3). This interpretation is consistent with the well-known previous findings by others; namely, while Ca^2+^ alone activates thin filaments by as much as ∼70% in cardiac muscle (Houmeida et al., 2010; Risi et al., 2017), the magnitude is only ∼20% in skeletal muscle, and strongly bound crossbridges need to be present for (near) maximal activation in skeletal muscle (Heeley et al., 2002, 2006, 2019). Because of the higher thermodynamic instability of Tm–Tn on F-actin, a higher fraction of crossbridges will be formed with cardiac thin filaments than with skeletal thin filaments on either type of myosin at temperatures lower than body temperature, resulting in higher apparent sensitivity to heating (Fig. 4). Clearly, future studies are needed to systematically investigate at the molecular level how thin filament structural changes and the ensuing myosin binding to actin occur by heating to the body temperature range at various concentrations of Ca^2+^ in skeletal versus cardiac muscle.

It is worthwhile noting that while a similar value of *Q*_10_ was obtained for cardiac thin filaments on both types of myosin at pCa 9 (3.3 and 5.1 on skeletal and β-cardiac myosin, respectively), it was ∼3.7 times greater for skeletal thin filaments on cardiac β-myosin than on skeletal myosin at pCa 9 (8.2 and 30 on skeletal and β-cardiac myosin, respectively). It can therefore be said that the temperature dependence of skeletal thin filaments (which is higher than that of cardiac thin filaments) depends dramatically on the type of myosin, presumably, the ATPase (i.e., ADP-release; see above) rate; namely, the faster the myosin’s ADP-release, the lower the value of *Q*_10_ in an in vitro motility assay. In addition, as reported by Matusovsky et al. (2019) based on a study using high-speed AFM, the structure of Tm is likely to have flexibility on cardiac thin filaments, and because of this flexibility, it may not be able to completely block the actomyosin interaction even under the relaxing condition. If a rise in temperature increases the flexibility of Tm as discussed above, the increased flexibility will likely hinder the inhibitory function of the Tm–Tn complex on the myosin-binding to actin.

It is worthwhile discussing the physiological significance of the present findings. We found that the *Q*_10_ was ∼1.6 times higher for skeletal thin filaments on skeletal myosin than that for cardiac thin filaments on β-cardiac myosin at pCa 9 (i.e., 8.2 versus 5.1; Figs. 2 and 3). The higher temperature dependence may allow fast skeletal muscle to contract quickly during activation once the cytosolic temperature increases during exercise or after a warm-up while saving energy at rest. In contrast, the lower temperature dependence may be beneficial for the homeostatic beating heart at partial activation to constantly eject blood from the ventricles during the entire life span. Therefore, depending on the physiological demands of the circumstances, mammalian striated muscle is fine-tuned to contract efficiently in the body temperature range. It will be important in future studies to systematically compare the temperature dependence of sarcomere shortening in isolated single myofibrils from three types of striated muscle, i.e., fast skeletal, slow skeletal, and cardiac muscles. Likewise, future studies are necessary to quantitatively investigate the temperature dependence of slow skeletal muscle proteins (myosin, Tm, and Tn).

## Supplementary Material

Table S1 provides a summary of the sliding velocities obtained in the present in vitro motility assay experiments on skeletal myosin at pCa 9.

Table S2 provides a summary of the sliding velocities obtained in the present in vitro motility assay experiments on skeletal myosin at pCa 5.

Table S3 provides a summary of the sliding velocity ratios at pCa 9/pCa 5 in the present in vitro motility assay experiments on skeletal myosin.

Table S4 provides a summary of the sliding velocities obtained in the present in vitro motility assay experiments on β-cardiac myosin at pCa 9.

Table S5 provides a summary of the sliding velocities obtained in the present in vitro motility assay experiments on β-cardiac myosin at pCa 5.

Table S6 provides a summary of the sliding velocity ratios at pCa 9/pCa 5 in the present in vitro motility assay experiments on β-cardiac myosin.

Table S7 provides a summary of the fractions of mobile filaments in the present in vitro motility assay experiments at pCa 9.

Table S8 provides a summary of the values of the temperature dependence obtained in our previous in vitro motility assay experiments (based on Ishii et al., 2019a).

## Data Availability

All relevant data, associated protocols, and materials are within the manuscript and its online supplemental material. If any additional information is needed, it will be available upon request from the corresponding authors.
