# Supplementary material for: Myosin and tropomyosin–troponin complementarily regulate thermal activation of muscles
Source: J Gen Physiol. 2023 Oct 23;155(12):e202313414. doi: 10.1085/jgp.202313414 (PMC10591409; doi:10.1085/jgp.202313414)
Supplement: Table S1 — provides a summary of the sliding velocities obtained in the present in vitro motility assay experiments on skeletal myosin at pCa 9. [file JGP_202313414_TableS1.docx]

**Table S1: Summary of the sliding velocities obtained in the present *in vitro* motility assay experiments on skeletal myosin at pCa 9.**

| Temperature  (°C) | F-actin (µm/s) | Skeletal TF (µm/s) | Cardiac TF (µm/s) | *P*  (F-actin vs. Skeletal TF) | *P*  (Skeletal vs. Cardiac TF) |
| --- | --- | --- | --- | --- | --- |
| 23 ± 1 | 4.3 **±** 0.06  (*n* = 185) | 0 | 1.4 **±** 0.25  (*n* = 10) | - | - |
| 26 ± 1 | - | 0.6 **±** 0  (*n* = 2) | 4.2 **±** 0.30  (*n* = 31) | - | - |
| 31 ± 0.5 | 11.7 **±** 0.40  (*n* = 24) | 2.1 **±** 0.46  (*n* = 7) | 7.4 **±** 1.17  (*n* = 10) | 1.80 × 10^-6^ | 2.77 × 10^-4^ |
| 32 ± 0.5 | 14.1 **±** 0.38  (*n* = 70) | 3.4 **±** 0.51  (*n* = 15) | 8.6 **±** 0.48  (*n* = 36) | 1.91 × 10^-6^ | 2.02 × 10^-6^ |
| 33 ± 0.5 | 14.8 **±** 0.23  (*n* = 101) | 4.9 **±** 0.31  (*n* = 59) | 9.3 **±** 0.47  (*n* = 50) | 1.86 × 10^-6^ | 1.86 × 10^-6^ |
| 34 ± 0.5 | 16.2 **±** 0.33  (*n* = 67) | 7.0 **±** 0.30  (*n* = 91) | 12.1 **±** 0.39  (*n* = 76) | 1.88 × 10^-6^ | 1.88 × 10^-6^ |
| 35 ± 0.5 | 16.7 **±** 0.36  (*n* = 111) | 7.1 **±** 0.32  (*n* = 83) | 13.7 **±** 0.37  (*n* = 91) | 1.92 × 10^-6^ | 1.92 × 10^-6^ |
| 36 ± 0.5 | 16.7 **±** 0.40  (*n* = 61) | 7.8 **±** 0.40  (*n* = 51) | 14.3 **±** 0.39  (*n* = 81) | 1.85 × 10^-6^ | 1.85 × 10^-6^ |
| 37 ± 0.5 | 17.1 **±** 0.34  (*n* = 70) | 10.1 **±** 0.33  (*n* = 132) | 15.1 **±** 0.39  (*n* = 75) | 1.91 × 10^-6^ | 1.91 × 10^-6^ |
| 38 ± 0.5 | 18.3 **±** 0.47  (*n* = 35) | 14.4 **±** 0.50  (*n* = 104) | 17.5 **±** 0.48  (*n* = 75) | 2.45 × 10^-5^ | 1.53 × 10^-6^ |
| 39 ± 0.5 | 18.5 **±** 0.46  (*n* = 45) | 18.1 **±** 0.54  (*n* = 105) | 21.2 **±** 0.44  (*n* = 71) | 0.82 | 4.31 × 10^-5^ |
| 40 ± 0.5 | 19.6 **±** 1.16  (*n* = 17) | 21.5 **±** 0.97  (*n* = 35) | 22.6 **±** 0.48  (*n* = 59) | 0.28 | 0.44 |

Temperature ranges indicated on left. Velocities expressed as mean ± SEM. *P* determined by Dunnett’s multiple comparison test. TF, thin filament.
