# Supplementary material for: Myosin and tropomyosin–troponin complementarily regulate thermal activation of muscles
Source: J Gen Physiol. 2023 Oct 23;155(12):e202313414. doi: 10.1085/jgp.202313414 (PMC10591409; doi:10.1085/jgp.202313414)
Supplement: Table S2 — provides a summary of the sliding velocities obtained in the present in vitro motility assay experiments on skeletal myosin at pCa 5. [file JGP_202313414_TableS2.docx]

**Table S2: Summary of the sliding velocities obtained in the present *in vitro* motility assay experiments on skeletal myosin at pCa 5.**

| Temperature  (°C) | F-actin (µm/s) | Skeletal TF (µm/s) | Cardiac TF (µm/s) | *P*  (F-actin vs. Skeletal TF) | *P*  (Skeletal vs. Cardiac TF) |
| --- | --- | --- | --- | --- | --- |
| 23 ± 1 | 4.8 **±** 0.05  (*n* = 269) | 7.2 **±** 0.12  (*n* = 206) | 8.4 **±** 0.05  (*n* = 356) | 2.04 × 10^-6^ | 2.04 × 10^-6^ |
| 26 ± 1 | - | 12.2 **±** 0.13  (*n* = 148) | 12.2 **±** 0.12  (*n* = 236) | - | - |
| 31 ± 0.5 | 14.4 **±** 0.28  (*n* = 45) | 18.1 **±** 0.59  (*n* = 21) | 16.8 **±** 0.44  (*n* = 29) | 1.84 × 10^-6^ | 0.074 |
| 32 ± 0.5 | 15.9 **±** 0.22  (*n* = 155) | 19.3 **±** 0.24  (*n* = 96) | 17.9 **±** 0.24  (*n* = 107) | 1.95 × 10^-6^ | 1.49 × 10^-4^ |
| 33 ± 0.5 | 17.5 **±** 0.22  (*n* = 192) | 20.2 **±** 0.24  (*n* = 107) | 18.5 **±** 0.23  (*n* = 130) | 1.98 × 10^-6^ | 6.46 × 10^-6^ |
| 34 ± 0.5 | 18.2 **±** 0.23  (*n* = 193) | 21.7 **±** 0.26  (*n* = 131) | 19.6 **±** 0.29  (*n* = 87) | 1.97 × 10^-6^ | 4.94 × 10^-6^ |
| 35 ± 0.5 | 18.9 **±** 0.25  (*n* = 206) | 22.0 **±** 0.31  (*n* = 103) | 20.6 **±** 0.29  (*n* = 103) | 1.97 × 10^-6^ | 5.08 × 10^-3^ |
| 36 ± 0.5 | 19.2 **±** 0.29  (*n* = 107) | 21.3 **±** 0.33  (*n* = 81) | 20.8 **±** 0.24  (*n* = 107) | 3.00 × 10^-6^ | 0.30 |
| 37 ± 0.5 | 20.4 **±** 0.36  (*n* = 75) | 22.8 **±** 0.25  (*n* = 165) | 21.6 **±** 0.22  (*n* = 120) | 1.96 × 10^-6^ | 1.74 × 10^-3^ |
| 38 ± 0.5 | 20.3 **±** 0.31  (*n* = 105) | 23.7 **±** 0.40  (*n* = 92) | 21.8 **±** 0.34  (*n* = 73) | 1.91 × 10^-6^ | 6.30 × 10^-4^ |
| 39 ± 0.5 | 21.4 **±** 0.45  (*n* = 59) | 24.1 **±** 0.39  (*n* = 82) | 21.8 **±** 0.33  (*n* = 74) | 5.03 × 10^-6^ | 2.93 × 10^-5^ |
| 40 ± 0.5 | 22.6 **±** 0.68  (*n* = 50) | 23.3 **±** 0.42  (*n* = 64) | 22.2 **±** 0.60  (*n* = 20) | 0.52 | 0.46 |

Temperature ranges indicated on left. Velocities expressed as mean ± SEM. *P* determined by Dunnett’s multiple comparison test. TF, thin filament.
