# Supplementary material for: Myosin and tropomyosin–troponin complementarily regulate thermal activation of muscles
Source: J Gen Physiol. 2023 Oct 23;155(12):e202313414. doi: 10.1085/jgp.202313414 (PMC10591409; doi:10.1085/jgp.202313414)
Supplement: Table S3 — provides a summary of the sliding velocity ratios at pCa 9/pCa 5 in the present in vitro motility assay experiments on skeletal myosin. [file JGP_202313414_TableS3.docx]

**Table S3: Summary of the sliding velocity ratios at pCa 9 / pCa 5 in the present *in vitro* motility assay experiments on skeletal myosin.**

| Temperature  (°C) | F-actin | Skeletal TF | Cardiac TF | *P*  (F-actin vs. Skeletal TF) | *P*  (Skeletal vs. Cardiac TF) |
| --- | --- | --- | --- | --- | --- |
| 23 ± 1 | 0.90 **±** 0.01 | 0 | 0.17 **±** 0.03 | - | - |
| 26 ± 1 | - | 0.05 **±** 0 | 0.34 **±** 0.02 | - | - |
| 31 ± 0.5 | 0.81 **±** 0.03 | 0.12 **±** 0.03 | 0.44 **±** 0.07 | 1.80 × 10^-6^ | 4.17 × 10^-4^ |
| 32 ± 0.5 | 0.89 **±** 0.02 | 0.17 **±** 0.03 | 0.48 **±** 0.03 | 1.91 × 10^-6^ | 2.18 × 10^-6^ |
| 33 ± 0.5 | 0.84 **±** 0.01 | 0.24 **±** 0.02 | 0.50 **±** 0.03 | 1.86 × 10^-6^ | 1.86 × 10^-6^ |
| 34 ± 0.5 | 0.89 **±** 0.02 | 0.32 **±** 0.01 | 0.62 **±** 0.02 | 1.88 × 10^-6^ | 1.88 × 10^-6^ |
| 35 ± 0.5 | 0.88 **±** 0.02 | 0.32 **±** 0.01 | 0.67 **±** 0.02 | 1.92 × 10^-6^ | 1.92 × 10^-6^ |
| 36 ± 0.5 | 0.87 **±** 0.02 | 0.37 **±** 0.02 | 0.69 **±** 0.02 | 1.85 × 10^-6^ | 1.85 × 10^-6^ |
| 37 ± 0.5 | 0.84 **±** 0.02 | 0.44 **±** 0.01 | 0.70 **±** 0.02 | 1.91 × 10^-6^ | 1.91 × 10^-6^ |
| 38 ± 0.5 | 0.91 **±** 0.02 | 0.61 **±** 0.02 | 0.81 **±** 0.02 | 1.87 × 10^-6^ | 1.87 × 10^-6^ |
| 39 ± 0.5 | 0.87 **±** 0.02 | 0.75 **±** 0.02 | 0.97 **±** 0.02 | 1.86 × 10^-3^ | 1.87 × 10^-3^ |
| 40 ± 0.5 | 0.87 **±** 0.05 | 0.92 **±** 0.04 | 1.02 **±** 0.02 | 0.57 | 0.058 |

Temperature ranges indicated on left. Velocity ratios expressed as mean ± SEM. *P* determined by Dunnett’s multiple comparison test. TF, thin filament.
