# Supplementary material for: Myosin and tropomyosin–troponin complementarily regulate thermal activation of muscles
Source: J Gen Physiol. 2023 Oct 23;155(12):e202313414. doi: 10.1085/jgp.202313414 (PMC10591409; doi:10.1085/jgp.202313414)
Supplement: Table S4 — provides a summary of the sliding velocities obtained in the present in vitro motility assay experiments on β-cardiac myosin at pCa 9. [file JGP_202313414_TableS4.docx]

**Table S4: Summary of the sliding velocities obtained in the present *in vitro* motility assay experiments on β-cardiac myosin at pCa 9.**

| Temperature  (°C) | F-actin (µm/s) | Skeletal TF (µm/s) | Cardiac TF (µm/s) | *P*  (F-actin vs. Skeletal TF) | *P*  (Skeletal vs. Cardiac TF) |
| --- | --- | --- | --- | --- | --- |
| 23 ± 1 | 0.4 **±** 0.01  (*n* = 132) | 0 | 0 | - | - |
| 31 ± 0.5 | 2.8 **±** 0.17  (*n* = 21) | 0 | 0.8 **±** 0.11  (*n* = 12) | - | - |
| 32 ± 0.5 | 3.3 **±** 0.12  (*n* = 52) | 0.2 **±** 0.02  (*n* = 10) | 0.8 **±** 0.05  (*n* = 57) | 1.88 × 10^-6^ | 5.79 × 10^-3^ |
| 33 ± 0.5 | 3.9 **±** 0.12  (*n* = 63) | 0.4 **±** 0.07  (*n* = 8) | 1.1 **±** 0.10  (*n* = 49) | 1.91 × 10^-6^ | 0.043 |
| 34 ± 0.5 | 4.4 **±** 0.13  (*n* = 68) | 1.2 **±** 0.22  (*n* = 20) | 1.3 **±** 0.06  (*n* = 105) | 1.85 × 10^-6^ | 0.90 |
| 35 ± 0.5 | 5.0 **±** 0.21  (*n* = 41) | 0.9 **±** 0.21  (*n* = 13) | 1.7 **±** 0.07  (*n* = 104) | 1.81 × 10^-6^ | 0.014 |
| 36 ± 0.5 | 4.9 **±** 0.49  (*n* = 8) | 1.0 **±** 0.09  (*n* = 26) | 1.5 **±** 0.09  (*n* = 64) | 1.80 × 10^-6^ | 4.19 × 10^-3^ |
| 37 ± 0.5 | 6.5 **±** 0.21  (*n* = 68) | 1.8 **±** 0.17  (*n* = 32) | 1.7 **±** 0.12  (*n* = 60) | 1.81 × 10^-6^ | 0.96 |
| 38 ± 0.5 | 7.4 **±** 0.27  (*n* = 36) | 2.2 **±** 0.14  (*n* = 84) | 2.1 **±** 0.09  (*n* = 98) | 1.87 × 10^-6^ | 0.73 |
| 39 ± 0.5 | 8.2 **±** 0.37  (*n* = 26) | 3.9 **±** 0.20  (*n* = 108) | 2.8 **±** 0.12  (*n* = 97) | 1.95 × 10^-6^ | 1.27 × 10^-5^ |
| 40 ± 0.5 | 9.5 **±** 0.42  (*n* = 30) | 6.2 **±** 0.26  (*n* = 63) | 4.1 **±** 0.20  (*n* = 74) | 1.82 × 10^-6^ | 1.83 × 10^-6^ |

Temperature ranges indicated on left. Velocities expressed as mean ± SEM. *P* determined by Dunnett’s multiple comparison test. TF, thin filament.
