# Supplementary material for: Myosin and tropomyosin–troponin complementarily regulate thermal activation of muscles
Source: J Gen Physiol. 2023 Oct 23;155(12):e202313414. doi: 10.1085/jgp.202313414 (PMC10591409; doi:10.1085/jgp.202313414)
Supplement: Table S5 — provides a summary of the sliding velocities obtained in the present in vitro motility assay experiments on β-cardiac myosin at pCa 5. [file JGP_202313414_TableS5.docx]

**Table S5: Summary of the sliding velocities obtained in the present *in vitro* motility assay experiments on β-cardiac myosin at pCa 5.**

| Temperature  (°C) | F-actin (µm/s) | Skeletal TF (µm/s) | Cardiac TF (µm/s) | *P*  (F-actin vs. Skeletal TF) | *P*  (Skeletal vs. Cardiac TF) |
| --- | --- | --- | --- | --- | --- |
| 23 ± 1 | 0.5 **±** 0.01  (*n* = 217) | 0.5 **±** 0.02  (*n* = 178) | 1.0 **±** 0.02  (*n* = 229) | 0.71 | 2.02 × 10^-6^ |
| 31 ± 0.5 | 3.9 **±** 0.23  (*n* = 18) | 3.7 **±** 0.27  (*n* = 17) | 5.5 **±** 0.22  (*n* = 18) | 0.80 | 1.76 × 10^-6^ |
| 32 ± 0.5 | 4.1 **±** 0.10  (*n* = 68) | 4.7 **±** 0.13  (*n* = 78) | 5.8 **±** 0.16  (*n* = 54) | 1.29 × 10^-3^ | 1.90 × 10^-6^ |
| 33 ± 0.5 | 4.7 **±** 0.12  (*n* = 89) | 5.3 **±** 0.12  (*n* = 109) | 6.4 **±** 0.14  (*n* = 62) | 6.94 × 10^-4^ | 1.99 × 10^-6^ |
| 34 ± 0.5 | 5.6 **±** 0.17  (*n* = 74) | 6.4 **±** 0.15  (*n* = 83) | 7.2 **±** 0.19  (*n* = 54) | 2.46 × 10^-3^ | 2.93 × 10^-3^ |
| 35 ± 0.5 | 6.2 **±** 0.15  (*n* = 116) | 6.9 **±** 0.13  (*n* = 166) | 7.4 **±** 0.14  (*n* = 105) | 1.26 × 10^-3^ | 0.017 |
| 36 ± 0.5 | 6.8 **±** 0.20  (*n* = 76) | 7.0 **±** 0.18  (*n* = 97) | 7.8 **±** 0.13  (*n* = 119) | 0.49 | 2.54 × 10^-3^ |
| 37 ± 0.5 | 7.1 **±** 0.19  (*n* = 61) | 8.2 **±** 0.18  (*n* = 118) | 8.3 **±** 0.14  (*n* = 121) | 1.29 × 10^-4^ | 0.83 |
| 38 ± 0.5 | 8.6 **±** 0.25  (*n* = 89) | 8.8 **±** 0.16  (*n* = 131) | 8.6 **±** 0.20  (*n* = 65) | 0.52 | 0.68 |
| 39 ± 0.5 | 9.6 **±** 0.25  (*n* = 88) | 9.9 **±** 0.19  (*n* = 97) | 8.5 **±** 0.29  (*n* = 26) | 0.54 | 5.57 × 10^-3^ |
| 40 ± 0.5 | 11.0 **±** 0.42  (*n* = 35) | 10.8 **±** 0.32  (*n* = 47) | 8.7 **±** 0.30  (*n* = 19) | 0.93 | 1.13 × 10^-3^ |

Temperature ranges indicated on left. Velocities expressed as mean ± SEM. *P* determined by Dunnett’s multiple comparison test. TF, thin filament.
