# Supplementary material for: Myosin and tropomyosin–troponin complementarily regulate thermal activation of muscles
Source: J Gen Physiol. 2023 Oct 23;155(12):e202313414. doi: 10.1085/jgp.202313414 (PMC10591409; doi:10.1085/jgp.202313414)
Supplement: Table S6 — provides a summary of the sliding velocity ratios at pCa 9/pCa 5 in the present in vitro motility assay experiments on β-cardiac myosin. [file JGP_202313414_TableS6.docx]

**Table S6: Summary of the sliding velocity ratios at pCa 9 / pCa 5 in the present *in vitro* motility assay experiments on β-cardiac myosin.**

| Temperature  (°C) | F-actin | Skeletal TF | Cardiac TF | *P*  (F-actin vs. Skeletal TF) | *P*  (Skeletal vs. Cardiac TF) |
| --- | --- | --- | --- | --- | --- |
| 23 ± 1 | 0.87 **±** 0.02 | 0 | 0 | - | - |
| 31 ± 0.5 | 0.73 **±** 0.05 | 0 | 0.15 **±** 0.02 | - | - |
| 32 ± 0.5 | 0.81 **±** 0.03 | 0.04 **±** 0.004 | 0.14 **±** 0.01 | 1.90 × 10^-6^ | 0.083 |
| 33 ± 0.5 | 0.83 **±** 0.03 | 0.08 **±** 0.01 | 0.17 **±** 0.02 | 1.91 × 10^-6^ | 0.17 |
| 34 ± 0.5 | 0.79 **±** 0.02 | 0.19 **±** 0.03 | 0.18 **±** 0.01 | 1.85 × 10^-6^ | 0.88 |
| 35 ± 0.5 | 0.80 **±** 0.03 | 0.13 **±** 0.03 | 0.22 **±** 0.01 | 1.81 × 10^-6^ | 0.047 |
| 36 ± 0.5 | 0.72 **±** 0.07 | 0.14 **±** 0.01 | 0.20 **±** 0.01 | 1.80 × 10^-6^ | 0.029 |
| 37 ± 0.5 | 0.92 **±** 0.03 | 0.21 **±** 0.02 | 0.20 **±** 0.01 | 1.81 × 10^-6^ | 0.95 |
| 38 ± 0.5 | 0.87 **±** 0.03 | 0.25 **±** 0.02 | 0.24 **±** 0.01 | 1.87 × 10^-6^ | 0.91 |
| 39 ± 0.5 | 0.86 **±** 0.04 | 0.40 **±** 0.02 | 0.33 **±** 0.01 | 1.88 × 10^-6^ | 0.031 |
| 40 ± 0.5 | 0.87 **±** 0.04 | 0.58 **±** 0.02 | 0.48 **±** 0.02 | 1.82 × 10^-6^ | 7.01 × 10^3^ |

Temperature ranges indicated on left. Velocity ratios expressed as mean ± SEM. *P* determined by Dunnett’s multiple comparison test. TF, thin filament.
