# Supplementary material for: Myosin and tropomyosin–troponin complementarily regulate thermal activation of muscles
Source: J Gen Physiol. 2023 Oct 23;155(12):e202313414. doi: 10.1085/jgp.202313414 (PMC10591409; doi:10.1085/jgp.202313414)
Supplement: Table S7 — provides a summary of the fractions of mobile filaments in the present in vitro motility assay experiments at pCa 9. [file JGP_202313414_TableS7.docx]

**Table S7: Summary of the fractions of mobile filaments in the present *in vitro* motility assay experiments at pCa 9.**

| Temperature  (°C) | Skeletal TF | Cardiac TF | Skeletal TF | Cardiac TF |
| --- | --- | --- | --- | --- |
|  | **Skeletal myosin** | | **β-Cardiac myosin** | |
| 23 ± 1 | 0/143  (0%) | 3/112  (2.7%) | 0/129  (0%) | 0/162  (0%) |
| 26 ± 1 | 2/83  (2.4%) | 22/84  (26%) | - | - |
| 31 ± 0.5 | 4/28  (14%) | 7/12  (58%) | 0/34  (0%) | 27/40  (68%) |
| 32 ± 0.5 | 20/57  (35%) | 98/103  (95%) | 9/71  (13%) | 60/72  (83%) |
| 33 ± 0.5 | 34/53  (64%) | 34/41  (83%) | 21/85  (25%) | 86/93  (92%) |
| 34 ± 0.5 | 45/53  (85%) | 49/51  (96%) | 29/52  (56%) | 80/85  (94%) |
| 35 ± 0.5 | 58/58  (100%) | 52/54  (96%) | 33/54  (61%) | 75/77  (97%) |
| 36 ± 0.5 | 38/39  (97%) | 46/47  (98%) | 38/49  (78%) | 43/43  (100%) |
| 37 ± 0.5 | 26/27  (96%) | 44/44  (100%) | 51/54  (94%) | 28/28  (100%) |
| 38 ± 0.5 | 29/29  (100%) | 35/35  (100%) | 47/47  (100%) | 31/31  (100%) |
| 39 ± 0.5 | 21/21  (100%) | 23/23  (100%) | 42/42  (100%) | 22/22  (100%) |
| 40 ± 0.5 | 11/11  (100%) | 17/17  (100%) | 16/16  (100%) | 20/20  (100%) |

Temperature ranges indicated on left. TF, thin filament.
