# Supplementary material for: Myosin and tropomyosin–troponin complementarily regulate thermal activation of muscles
Source: J Gen Physiol. 2023 Oct 23;155(12):e202313414. doi: 10.1085/jgp.202313414 (PMC10591409; doi:10.1085/jgp.202313414)
Supplement: Table S8 — provides a summary of the values of the temperature dependence obtained in our previous in vitro motility assay experiments (based on Ishii et al., 2019a). [file JGP_202313414_TableS8.docx]

**Table S8: Summary of the values of the temperature dependence obtained in our previous *in vitro* motility assay experiments (based on Ishii et al*.*, 2019a).**

| Myosin type | Thin filament type | pCa | *Q*_10_ | *E*_a_ (kJ/mol) |
| --- | --- | --- | --- | --- |
| Skeletal | F-actin | 9 | 1.6 ± 0.05 | 36.9 ± 2.5 |
|  |  | 5 | 2.0 ± 0.3 | 54.3 ± 10.3 |
|  | Cardiac | 9 | 4.8 ± 1.5 | 126 ± 24 |
|  |  | 5 | 1.4 ± 0.1 | 29.6 ± 6.8 |
| β-cardiac | F-actin | 9 | 3.4 ± 0.5 | 99.2 ± 12.3 |
|  |  | 5 | 3.2 ± 0.7 | 93.6 ± 16.4 |
|  | Cardiac | 9 | 5.3 ± 2.8 | 135 ± 42 |
|  |  | 5 | 2.1 ± 0.07 | 60.1 ± 2.6 |

The values of *Q*_10_ and *E*_a_ for fast skeletal myosin and those for β-cardiac myosin were recalculated from the sliding velocities between 34 and 41°C and between 37 and 40°C, respectively, based on the data reported in Ishii et al*.*, 2019a (see **MATERIALS AND METHODS**). Data expressed as mean ± SEM.
